# Impacts of Circadian Gene *Period2* Knockout on Intestinal Metabolism and Hepatic Antioxidant and Inflammation State in Mice

**DOI:** 10.1155/2022/7896371

**Published:** 2022-07-19

**Authors:** Yongkang Zhen, Zanna Xi, Liangyu Hu, Yifei Chen, Ling Ge, Wenjun Wei, Juan J. Loor, Qingyong Yang, Mengzhi Wang

**Affiliations:** ^1^College of Animal Science and Technology, Yangzhou University, Yangzhou, Jiangsu, China; ^2^State Key Laboratory of Sheep Genetic Improvement and Healthy Production, Xinjiang Academy of Agricultural Reclamation Sciences, Shihezi, Xinjiang, China; ^3^Human and Animal Physiology, Wageningen University & Research, 6708 WD Wageningen, Netherlands; ^4^Mammalian Nutrition Physiology Genomics, Department of Animal Sciences and Division of Nutritional Sciences, University of Illinois, Urbana, IL, USA

## Abstract

The period circadian regulator 2 (*Per2*) gene is important for the modulations of rhythmic homeostasis in the gut and liver; disruption will cause metabolic diseases, such as obesity, diabetes, and fatty liver. Herein, we investigated the alterations in intestinal metabolic and hepatic functions in *Per2* knockout (*Per2*^−/−^, KO) and wild-type (*Per2*^+/+^, WT) mice. Growth indices, intestinal metabolomics, hepatic circadian rhythms, lipid metabolism, inflammation-related genes, antioxidant capacity, and transcriptome sequencing were performed after euthanasia. Data indicated that KO decreased the intestinal concentrations of amino acids such as *γ*-aminobutyric acid, aspartic acid, glycine, L-allothreonine, methionine, proline, serine, and valine while it increased the concentrations of carbohydrates such as cellobiose, D-talose, fucose, lyxose, and xylose compared with WT. Moreover, the imbalance of intestinal metabolism further seemed to induce liver dysfunction. Data indicated that *Per2* knockout altered the expression of hepatic circadian rhythm genes, such as *Clock*, *Bmal1*, *Per1*, *Per3*, *Cry1*, and *Cry2*. KO also induced hepatic lipid metabolism, because of the increase of liver index and serum concentrations of low-density lipoprotein, and the upregulated expression of *Pparα*, *Cyp7a1*, and *Cpt1*. In addition, KO improved hepatic antioxidant capacity due to the increase activities of SOD and GSH-Px and the decrease in concentrations of MDA. Lastly, KO increased the relative expression levels of hepatic inflammation-related genes, such as *Il-1β*, *Il-6*, *Tnf-α*, *Myd88*, and *Nf-κB p65*, which may potentially lead to hepatic inflammation. Overall, *Per2* knockout induces gut metabolic dysregulation and may potentially trigger alterations in hepatic antioxidant and inflammation responses.

## 1. Introduction

Circadian rhythms are defined as the cyclical changes in physiology, metabolism, behavior, and circulation according to a 24 h cycle in the life of mammals regulated by the periodic expression of a series of circadian clock genes [1, 2]. At present, many circadian clock genes have been identified in animals including period circadian clock 1/2/3 (*Per1/2/3*), cryptochrome 1/2 (*Cry1/2*), brain and muscle ARNT-like 1 (*Bmal1*), and circadian locomotor output cycle kaput (*Clock*) [3, 4]. A central circadian clock located in the suprachiasmatic nucleus (SCN) coordinates the oscillation of the peripheral circadian clock, while the intrinsically photosensitive retinal ganglion cells (ipRGCs) send light signals into SCN to guide the central clock to periodically oscillate and output rhythmic behaviors consistent with daily changes [5]. The circadian clock is produced by the transcriptional-translational feedback loops (TTFLs) of circadian genes [6, 7]. Mechanistically, the TTFLs involve events after the heterodimer formation by Clock and Bmal1 proteins that lead to activation of transcription and translation of *Per* and *Cry*. The Per and Cry proteins then undergo nuclear translocation and inhibit the formation of heterodimer, thereby inhibiting transcription [8].

Period circadian clock 2, as an important core circadian gene, is mainly expressed in the brain and spinal cord of the central nervous system, as well as in the surrounding organs such as the intestine, skin, and stomach; the mutation in *Per2* will shorten the circadian oscillation and inhibit the TTFL axis according to some previous reports [9–11]. Circadian genes are also involved in a variety of metabolic processes [12, 13]. For example, *PER2* is associated with abdominal obesity, psycho-behavioral factors, and attrition in the dietary treatment of obesity in humans [14]. In addition, *Per2* is closely related to glucose metabolism and myocardial function in mice [15]. Another study has shown that the deletion of *Per2* causes glucocorticoid imbalance and leads to circadian eating disorders, such as night-eating syndrome and obesity in mice [16, 17].

Rhythm disorders will also lead to imbalances in homeostasis of intestinal microbes and their metabolites [18]. It has been reported that *Per2* gene can interact with gut microbial metabolites; for example, the circadian oscillation of *Per2* in peripheral tissues can be modulated by 3-(4-hydroxyphenyl) propionic acid and 3-phenylpropionic acid which produced by *Clostridium sporogenes* [19]. We previously found that *Per2* knockout increased the relative abundance of Lachnospiraceae family and Ruminococcaceae family in the large intestine and further increased the concentration of butyrate [20]. Moreover, *Per2* is also associated with hepatic functions; for example, *Per2* knockout mice showed more severe liver fibrosis, cholestasis, or infarction under toxic conditions than wild-type mice [21]. It is also noteworthy to highlight that liver-specific deletion of *Per2* abolished daily food anticipation by regulating the production of *β*-hydroxybutyrate [22]. However, few studies have reported the impacts of *Per2* on global microbial metabolism in the large intestine, and it is still unknown whether these impacts could mediate changes in hepatic functions.

In this study, we hypothesized that a disorederd circadian rhythm caused by *Per2* knockout could potentially destroy intestinal metabolic homeostasis and induce negative impacts on hepatic functions, such as inflammation. This hypothesis is based on the fact that *Per2* is one of the core circadian clock genes and is closely related to intestinal and hepatic metabolic functions [15]. To address the hypothesis, *Per2* knockout (KO) and wild-type (WT) mice were managed under a regular light-dark cycle to study the impacts on intestinal metabolism using metabolomics and the hepatic circadian rhythms, lipid metabolism, inflammation, and antioxidant capacity using real-time PCR, biochemical kits, and transcriptome sequencing.

## 2. Materials and Methods

### 2.1. Ethics Statement

All animal experiments were performed according to the ethical policies and procedures approved by the Animal Care and Use Committee of Yangzhou University, Jiangsu, China (Approval no. SYXK (Su) 2017-0044).

### 2.2. Mouse Management and Experimental Design

The *Per2* knockout homozygous (*Per2^−/−^*) mice were used in our study. Firstly, heterozygous (*Per2*^+/-^) mice were generated by Biocytogen Pharmaceuticals (Beijing, China) using CRISPR/Cas9 technology. The C57BL/6N strain was used, and all mice were housed in a specific pathogen-free facility. Briefly, the candidate sgRNAs, respectively, located in the intron 4 and intron 22 of *Per2* were screened with the CRISPR design tool (http://www.sanger.ac.uk/htgt/wge/). The selected RNAs were then determined according to on-target activity use UCATM (Universal CRISPR Activity Assay). The Cas9 mRNA and sgRNAs were coinjected into the cytoplasm of one-cell stage fertilized eggs (C57BL/6N). The injected zygotes were transferred into oviducts of Kunming pseudopregnant females to generate F0 mice. The F0 mice with expected genotype, verified by tail genomic DNA PCR and sequencing, were mated with C57BL/6N mice to establish germline-transmitted F1 heterozygous (*Per2*^+/-^) mice. These F1 mice were further genotyped by tail genomic PCR and DNA sequencing.

Mice were then sent to nonhuman primate research platform, Chinese Academy of Science (Suzhou, China), to obtain a sufficient number of homozygous mice (*Per2^−/−^*, KO) and wild-type mice (*Per2^+/+^*, WT). The 8-week-old KO and WT mice were in good health, similar in body size and initial body weight (*n* = 6). Each mouse was managed in a single cage with an environmentally controlled warehouse that allowed for manipulating the light-dark cycle of 12 h light and 12 h dark. The lights were turned on at 6 AM (ZT0) and turned off at 6 PM (ZT12).

Composition of the commercial maintenance pellet feed consisted of corn, soybean, wheat, chicken meal, fish meal, and vegetable oil. The trough, drinking fountain, and bedding were changed weekly. The experimental period was 42 days and was divided into 2 stages. The first stage lasted 28 days and was used for the acclimatization of the diets and light-dark cycle, and the next stage lasted 14 days and was used for formal trial under strict environmental control conditions.

All mice were fasted for 24 h before sampling, and the body weight was recorded. Blood was collected from the retroorbital sinus for measurement of serum biochemical indices. The KO and WT mice were then anesthetized with ether and euthanized by spinal dislocation during ZT4-ZT6 (*n* = 3) and ZT16-18 (*n* = 3). The intestinal contents were stripped along the outer wall of the colon for metabolomics analysis. The liver, heart, thymus, spleen, and other organs were isolated and weighed after blotting excess blood, and samples immediately snap-frozen in liquid nitrogen before storage at -80°C until analysis. The organ index was calculated following a previously described equation [23].

### 2.3. Detection of Serum Indicators

Blood samples were centrifuged at 4, 000 rpm and 4°C for 10 min to collect the serum. Then, the serum was sent to Yangzhou Medical Testing Center (Jiangsu, China) for the measurement of serum indicators.

### 2.4. Total RNA Extraction and Real-Time PCR (RT-PCR)

Total RNA was extracted from hepatic tissue using the FastPure Cell/Tissue Total RNA Isolation Kit V2 (RC112, Vazyme, Nanjing, China). Concentration and purity of total RNA were determined with a NanoDrop spectrophotometer (Thermo Fisher Scientific, Waltham, MA, USA). Reverse transcription referred to FastKing gDNA Dispelling RT Super Mix (TIANGEN, Beijing, China). Reverse transcription reaction system was as follows: RT Mix, 4 *μ*L; total RNA, 1, 000 ng; and RNase-free ddH_2_O to make the volume of 20 *μ*L. Reaction procedure was set as 42°C for 15 min and 95°C for 3 min following the manufacturer's instructions.

The reverse transcription gDNA samples were used as templates for real-time PCR (RT-PCR) using the 2× TSINGKE Master qPCR Mix (SYBR Green I) (TSE201, Tsingke, Beijing, China) in an ABI7500 (Thermo Fisher) sequence detector. The reaction system for PCR was as follows: qPCR Mix 10 *μ*L, forward primer 0.8 *μ*L (10 *μ*M), reverse primer 0.8 *μ*L (10 *μ*M), 50× ROX Reference Dye 0.4 *μ*L, and ddH_2_O up to 20 *μ*L. PCR reaction procedure was 95°C for 60 s, 40 cycles of 95°C for 10 s, and 60°C for 30 s, ultimately tested at 95°C for 15 s, 60°C for 60 s, 95°C for 30 s, and 60°C for 15 s. The standard curve method and QuantStudio™ 7 Flex Real-Time PCR Software (Applied Biosystems, CA) were used for data analysis. Results were analyzed using 2^−∆∆Ct^ method [24]. Specific primers used for RT-PCR were shown in Table S1.

### 2.5. Hepatic Antioxidant Capacity Analysis

Hepatic tissues were immediately extracted and homogenized with extraction solution on ice for antioxidant capacity analysis. The homogenized samples were centrifuged at 8000 rpm at 4°C for 10 min to collect the supernatant. Protein concentrations were determined using total protein quantitative assay kit (A045-2, Nanjing Jiancheng Bioengineering Institute, Nanjing, China). The activity of glutathione peroxidase (GSH-Px) was determined using colorimetric test kit (A005-1, Jiancheng). The activities of superoxide dismutase (SOD) (BC5165, Beijing Solarbio Science & Technology Co., Ltd., Beijing, China), catalase (CAT) (BC0205, Solarbio), glutathione (GSH) (BC1175, Solarbio), and malondialdehyde (MDA) (BC0025, Solarbio) were assayed using biochemical kits following the manufacturer's protocols.

### 2.6. Metabolite Extraction and Nontargeted Metabolomics

Fifty-milligram intestinal content mixture was ground with liquid nitrogen for metabolite extraction. The homogenate was resuspended with a 450 *μ*L extraction solution and vortexed. Samples were then centrifuged at 12, 000 rpm and 4°C for 20 min. A 350 *μ*L of supernatant was pipetted into a 1.5 mL EP tube and mixed 80 *μ*L of each sample into the QC samples [25]. The supernatant was then dried under vacuum and 80 *μ*L of MOX reagent (O-methoxyamine HCl, dissolved in 20 mg/mL pyridine) added, vortexed, and incubated at 80°C for 30 min. A 100 *μ*L of BSTFA (containing 1% TMCS) was added, vortexed, and incubated at 70°C for 90 min. Lastly, a 10 *μ*L of FAME (dissolved in chloroform) was added after cooling. The Agilent 7890 gas chromatograph (Agilent Technologies, USA) was used for GC-TOF-MS analysis with a Agilent DB-5MS (30 m × 250 *μ*m × 0.25 *μ*m, J&W Scientific, USA) capillary column following the manufacturer's instructions [26, 27].

The raw data files generated by GC-TOF-MS were further processed with the ChromaTOF software (V4.3x, LECO) to perform peak extraction, baseline correction, and peak alignment. The metabolites were matched using LECO/Fiehn Metabolomics Library Database [28] and annotated using KEGG (https://www.genome.jp/kegg/pathway.html) and HMDB (https://hmdb.ca/metabolites) databases. Principal component analysis (PCA) and partial least square discriminant analysis (PLS-DA) were performed using the SIMCA-P software (version 14.1, Umetrics, Umea, Sweden). Analysis of variance (ANOVA) was conducted using the SPSS software (version 13.0) to calculate statistical significance (*P* value). Differential metabolites were considered as VIP > 1 and *P* < 0.05. Volcano plots were used to filter metabolites of interest based on log_2_ (fold change) and -log_10_ (*P* value). The functions and metabolic pathways were enriched in KEGG database with MetaboAnalyst 5.0 website (https://www.metaboanalyst.ca/MetaboAnalyst/ModuleView.xhtml) [29].

### 2.7. Hepatic Transcriptome Sequencing and Data Processing

The raw sequencing data of hepatic tissues in *Per2* knockout and wild-type mice were downloaded from [22] (https://www.ncbi.nlm.nih.gov/bioproject/PRJNA281172) (Exp1) and [30] (https://www.ncbi.nlm.nih.gov/geo/query/acc.cgi?acc=GSE156450) (Exp2).

The downloaded data from Exp1 (*n* = 3 for each group) and Exp2 (*n* = 2 for each group) were used for downstream analysis. The Fastp software (v0.23.1) [31] was used to perform quality control on the raw data and obtain clean data. The HISAT2 software (v2.1.0) [32] was used to align the obtained clean data to the reference genome (Mus musculus genome, GRCm39). The SAMtools software (v.1.10) was used to sort and convert the SAM files to BAM format [33]. The StringTie (v2.2.1) software was used to assemble and quantify the transcripts and genes based on read counts [32]. Lastly, the expression levels of all mRNA were estimated using DESeq2 package (1.36.0) in the R (v4.2) software. The differential expression genes (DEGs) of Exp1 and Exp2 were selected according to a threshold of *P* < 0.05 and |log_2_fold change| > 1; then, the DEGs shared by Exp1 and Exp2 were considered for further analysis. Functional enrichment was analyzed and visualized using KEGG database. The hierarchical clustering was generated using Pheatmap package (v.1.0.12).

### 2.8. Statistical Analysis

All indicators except for the metabolomics and transcriptome sequencing data were subjected to the Students' *t*-test using the SPSS 13.0 (SPSS, Inc., Chicago, IL, US) software. The GraphPad Prism (v6.0) software was used to draw the histograms. Results were represented as mean ± SEM (∗ denotes *P* < 0.05, significant difference; ∗∗ or ∗∗∗ denotes *P* < 0.01 or *P* < 0.001, extremely significant difference).

## 3. Results

### 3.1. Relative Expression Levels of Hepatic Circadian Rhythm Genes

**Figure 1:**
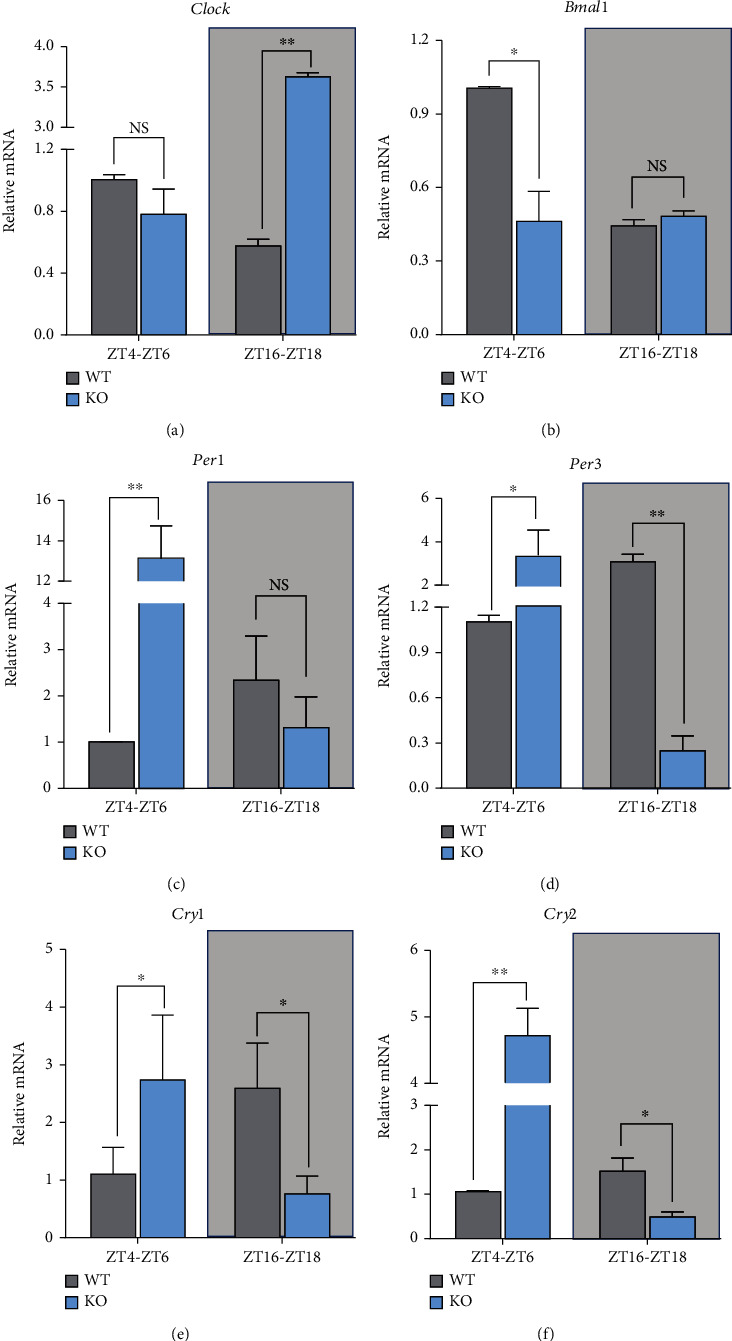
The relative expression levels of hepatic circadian rhythm genes in KO and WT mice. WT: wild-type (Period2^+/+^) mice; KO: *Per2* gene knockout (Period2^−/−^) mice. Representative charts of the relative expression levels of *Clock* (a), *Bmal1* (b), *Per1* (c), *Per3* (d), *Cry1* (e), and *Cry2* (f) were determined in liver tissues of *Per2* knockout and wild-type mice during ZT4-ZT6 and ZT16-ZT18 by RT-PCR method. ∗, *P* < 0.05, significant difference; ∗∗ or ∗∗∗, *P* < 0.01 or *P* < 0.001, extremely significant difference; NS, *P* > 0.05, without a difference. Data were shown as mean ± SEM; *n* = 3 biological replicates.

Compared with WT, KO significantly decreased the relative expression level of *Bmal1* (Figure 1(b)) while it increased the relative expression levels of *Per1* (Figure 1(c)), *Per3* (Figure 1(d)), *Cry1* (Figure 1(e)), and *Cry2* (Figure 1(f)) during ZT4-ZT6 (*P* < 0.05 or *P* < 0.01) but had no impacts on the relative expression level of *Clock* (Figure 1(a)) (*P* > 0.05) in the liver. In terms of the relative expression levels of circadian rhythm genes during ZT16-ZT18, KO significantly increased the relative expression level of *Clock* (Figure 1(a)) while it decreased the relative expression levels of *Per3* (Figure 1(d)), *Cry1* (Figure 1(e)), and *Cry2* (Figure 1(f)) (*P* < 0.05 or *P* < 0.01) but had no impacts on the relative expression levels of *Bmal1* (Figure 1(b)) and *Per1* (Figure 1(c)) compared with WT (*P* > 0.05) in the liver.

### 3.2. Serum Indicators, Organ Weights, and Relative Expression Levels of Hepatic Lipid Metabolism-Related Genes

**Figure 2:**
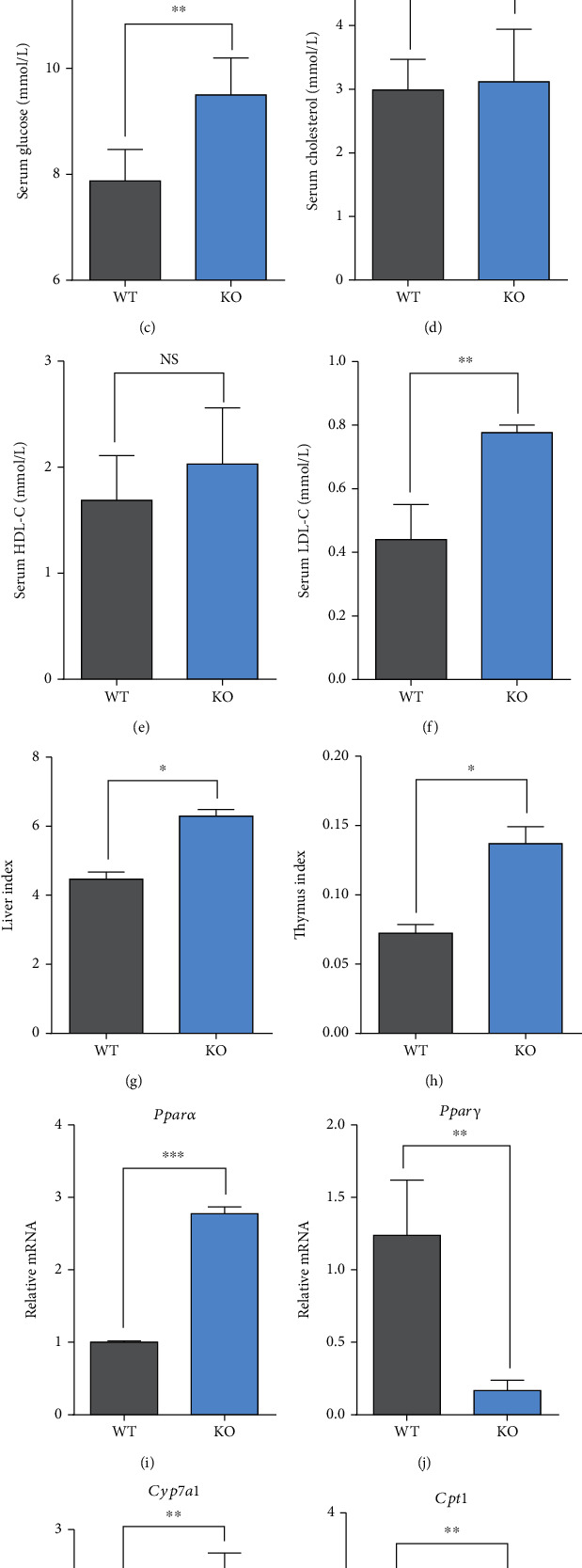
Serum indicators, organ weights, and the relative expression levels of hepatic lipid metabolism-related genes in KO and WT mice. WT: wild-type (Period2^+/+^) mice; KO: *Per2* gene knockout (Period2^−/−^) mice. Representative charts of the serum concentrations of ALT (a), AST (b), glucose (c), cholesterol (d), HDL-C (e), LDL-C (f), liver index (g), and thymus index (h) were measured in *Per2* knockout and wild-type mice. The relative expression levels of *Pparα* (i), *Pparγ* (j), *Cyp7a1* (k), and *Cpt1* (I) were determined in liver tissues of *Per2* knockout and wild-type mice by RT-PCR method. ∗, *P* < 0.05, significant difference; ∗∗ or ∗∗∗, *P* < 0.01 or *P* < 0.001, extremely significant difference; NS, *P* > 0.05, without a difference. Data were shown as mean ± SEM; *n* = 6 biological replicates.

Compared with WT, KO significantly increased the concentrations of serum glucose (Figure 2(c)) and low-density lipoprotein (LDL-C) (Figure 2(f)) (*P* < 0.01) but had no impact on the concentrations of serum alanine aminotransferase (ALT) (Figure 2(a)), aspartate aminotransferase (AST) (Figure 2(b)), cholesterol (Figure 2(d)), and high-density lipoprotein (HDL-C) (Figure 2(e)) (*P* > 0.05). Moreover, KO increased liver index (Figure 2(g)) and thymus index (Figure 2(h)) compared with WT (*P* < 0.05). In terms of hepatic lipid metabolism-related genes, KO increased the relative expression levels of *Pparα* (Figure 2(i)), *Cyp7a1* (Figure 2(k)), and *Cpt1* (Figure 2(l)) while it decreased the relative expression level of *Pparγ* (Figure 2(j)) compared with WT (*P* < 0.01 or *P* < 0.001).

### 3.3. Activities of Hepatic Antioxidant Capacity Indicators

**Figure 3:**
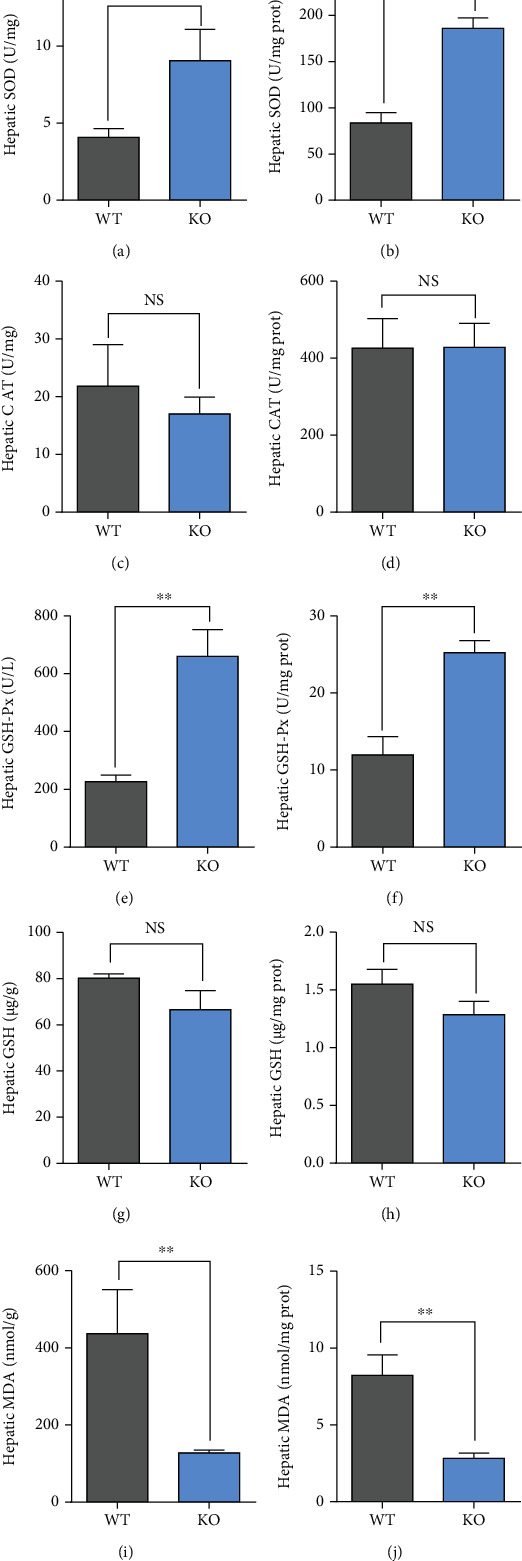
The activities of hepatic antioxidant capacity indicators in KO and WT mice. WT: wild-type (Period2^+/+^) mice; KO: *Per2* gene knockout (Period2^−/−^) mice. Representative charts of the activities of superoxide dismutase (a, b), catalase (c, d), glutathione peroxidase (e, f), glutathione (g, h), and the concentration of malondialdehyde (i, j) were determined in liver tissues of *Per2* knockout and wild-type mice as a function of liver weight and liver protein. ∗, *P* < 0.05, significant difference; ∗∗ or ∗∗∗, *P* < 0.01 or *P* < 0.001, extremely significant difference; NS, *P* > 0.05, without a difference. Data were shown as mean ± SEM; *n* = 6 biological replicates.

Compared with WT, the activity of hepatic SOD was significantly upregulated in KO mice both as a function of liver weight (Figure 3(a)) and liver protein (Figure 3(b)) (*P* < 0.01). Besides, the activity of hepatic GSH-Px was also significantly upregulated in KO mice as a function of liver weight (Figure 3(e)) and liver protein (Figure 3(f)) (*P* < 0.01). However, the activities of hepatic CAT and GSH as a function of liver weight (Figure 3(c) and Figure 3(g)) and liver protein (Figure 3(d) and Figure 3(h)) between KO and WT did not differ (*P* > 0.05). Lastly, the concentration of hepatic MDA was significantly downregulated in KO mice as a function of liver weight (Figure 3(i)) and liver protein (Figure 3(j)) (*P* < 0.01).

### 3.4. Overview of Intestinal Metabolomics Profiles and Identification of Significantly Different Metabolites

**Figure 4:**
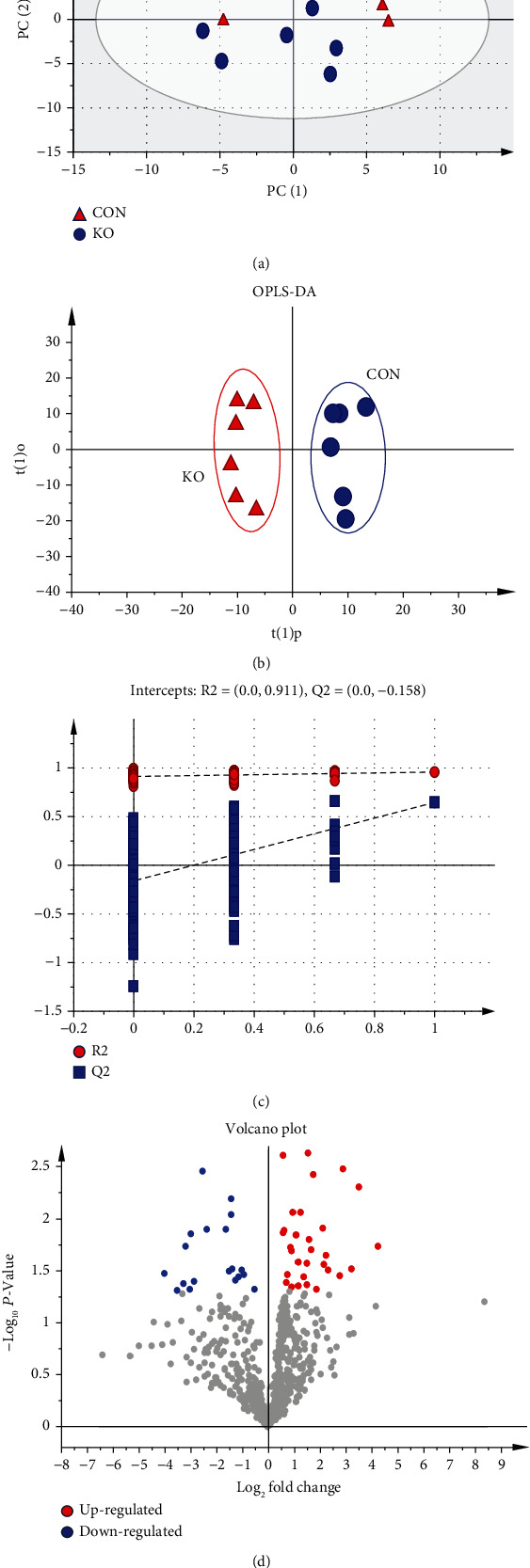
Construction of the PCA and OPLS-DA metabolomics model in the intestinal content of KO and WT mice. WT: wild-type (Period2^+/+^) mice; KO: *Per2* gene knockout (Period2^−/−^) mice. (a) The PCA plot based on GC-TOF-MS analysis in KO and WT mice. (b) The OPLS-DA plot based on GC-TOF-MS analysis in KO and WT mice. (c) The OPLS-DA corresponding validation plots based on 200 times permutation tests. (d) Volcano plots of significantly differential metabolites. Red dots represent upregulated; blue dots represent downregulated metabolites.

Multivariate statistical methods were applied to analyze metabolomics data based on GC-TOF-MS. First, PCA score chart was used to show the distribution of the original data and indicated that metabolites in the intestinal content of KO and WT mice showed slight separation on the PC2 axis (Figure 4(a)). To further illustrate the contribution of KO to the classification and differentiation of metabolites, an OPLS-DA score chart was used to clarify the metabolic pattern. Data indicated that the metabolites in the intestinal contents of KO and WT mice were significantly separated on the PC1 axis (Figure 4(b)), which indicated that KO significantly altered the metabolism mode of intestinal content. In addition, a permutation test was used to test the reliability of the model (Figure 4(c)); the *R*^2^*Y* and *Q*^2^ intercept values were 0.911 and -0.158, respectively. The *Q*^2^ intercept values were less than zero, indicating that the OPLS-DA model was reliable and could be used for downstream analysis [27, 34, 35].

**Figure 5:**
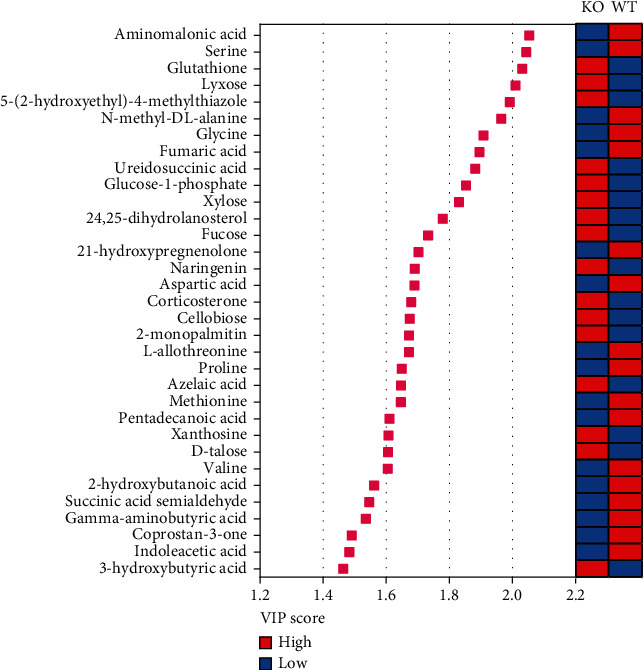
The identification of significantly different metabolites. WT: wild-type (Period2^+/+^) mice; KO: *Per2* gene knockout (Period2^−/−^) mice. Variable importance in projection (VIP) scores of the differential metabolites obtained from the OPLS-DA models. Red boxes represent high concentration, and blue boxes represent low concentration.

Metabolites with VIP > 1.0 and *P* < 0.05 were selected as differentially altered metabolites [36]. The volcano chart of differentially altered metabolites revealed a total of 50 between KO and WT mice (Figure 4(d)). Among those, 30 were upregulated while 20 were downregulated (Table S2 and Figure 4(d)) (analyte plus number represented unrecognized metabolites, and unknown represented unknown metabolites). There were 33 metabolites accurately identified. Differentially sorted according to the VIP value mainly included aminomalonic acid, serine, glutathione, lyxose, 5-(2-hydroxyethyl)-4-methylthiazole, N-methyl-DL-alanine, and glycine (Figure 5).

### 3.5. Characterization and Functional Analysis of Key Metabolite Pathways

**Figure 6:**
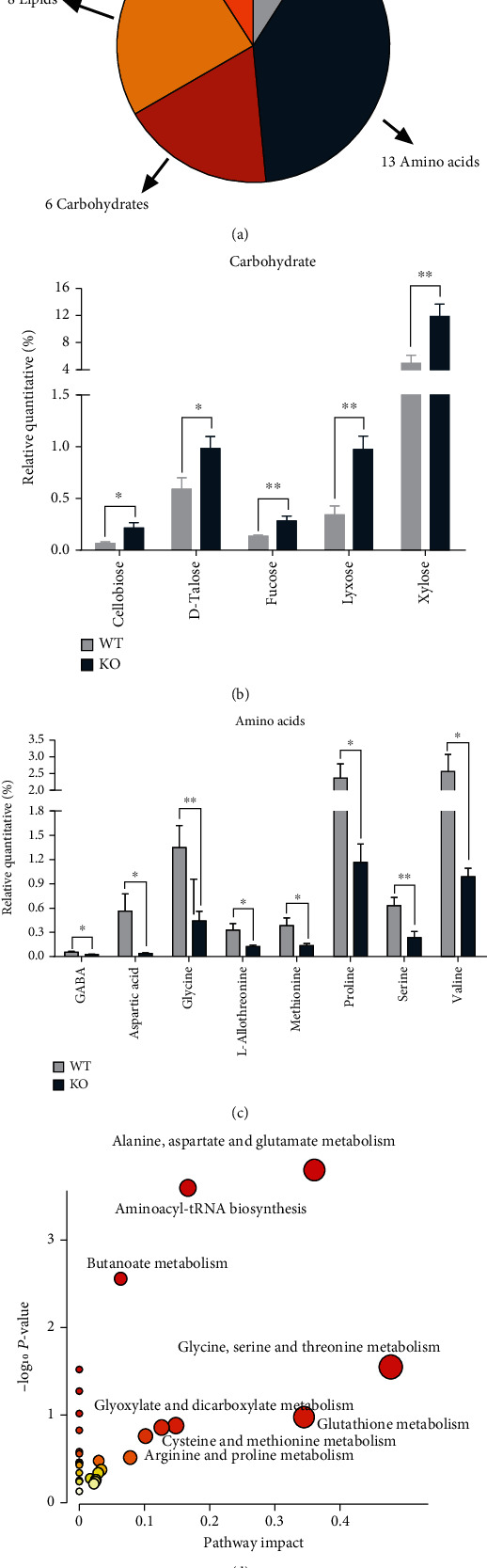
The characterization and functional analysis of key metabolites. WT: wild-type (Period2^+/+^) mice; KO: *Per2* gene knockout (Period2^−/−^) mice. (a) The classification of significantly differential metabolites. (b) Bar chart of the significant differential metabolites belonged to amino acids. (c) Bar chart of the significant differential metabolites belonged to carbohydrates. (d) Significant metabolic pathway maps enriched in KEGG database. Large sizes and dark colors represent high pathway impact and major pathway enrichment, respectively.

The classification of differential metabolites is shown in Figure 6(a). Among those, 13 metabolites belonged to amino acids, and 6 metabolites belonged to carbohydrate, while 8 metabolites belonged to lipids and 3 metabolites belonged to organic acids, but 3 of total 33 metabolites were unclassified. The differentially altered metabolites were further analyzed to study the effect of *Per2* knockout on amino acid and carbohydrate metabolism in the hindgut of mice. Data indicated that the majority of differentially altered amino acids in KO mice were significantly lower than those in WT (*P* < 0.05 or *P* < 0.01). These mainly included *γ*-aminobutyric acid (GABA), aspartic acid, glycine, L-allothreonine, methionine, proline, serine, and valine (Figure 6(b)), while most differentially altered carbohydrates were upregulated compared with WT. These included cellobiose, D-talose, fucose, lyxose, and xylose (*P* < 0.05) (Figure 6(c)).

The enrichment results of specific metabolites *via* KEGG database are shown in Figure 6(d) and Table S3. Different metabolites were mainly enriched in “alanine, aspartate, and glutamate metabolism” pathway of aspartic acid, succinate semialdehyde, GABA, fumaric acid, and carbamoyl-aspartic acid; “aminoacyl-tRNA biosynthesis” pathway of glycine, aspartic acid, serine, methionine, valine, and proline; “butanoate metabolism” pathway of 3-hydroxybutyric acid, GABA, and succinate semialdehyde; and “glycine, serine, and threonine metabolism” pathway of serine, glycine, and L-allothreonine, via KEGG database.

### 3.6. Hepatic Transcriptome Sequencing Profiles and Pathway Enrichment

**Figure 7:**
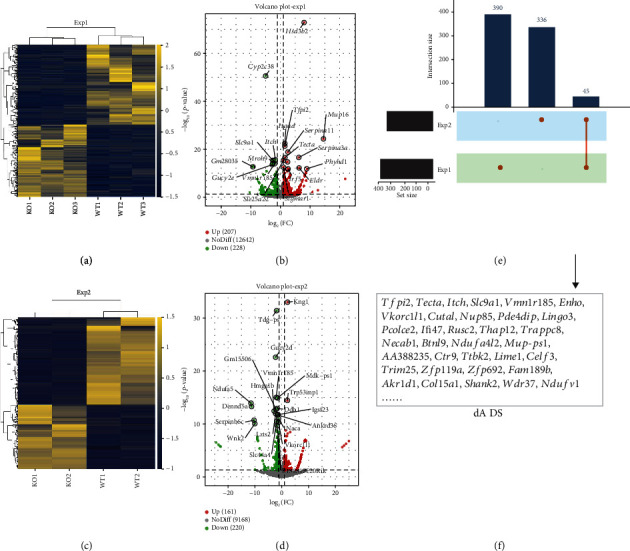
Hepatic transcriptome sequencing profiles in KO and WT mice. WT: wild-type (Period2^+/+^) mice; KO: *Per2* gene knockout (Period2^−/−^) mice. The differentially expressed genes (DEGs) were screened out by setting *P* < 0.05 and |log_2_fold change| > 1 as threshold. (a) The heat map of the DEGs in Exp1. (b) The volcano plot of the DEGs in Exp1; red dots represent upregulated, and green dots represent downregulated. (c) The heat map of the DEGs in Exp2. (d) The volcano plot of the DEGs in Exp2; red dots represent upregulated, and green dots represent downregulated. (e) Bar chart of the number of DEGs in Exp1, Exp2, and their coexpressed genes. (f) The list of coexpressed DEGs in Exp1 and Exp2.

We next investigated the alterations in hepatic functions in WT and KO mice using transcriptome sequencing. Data indicated that a total number of 435 DEGs were selected in Exp1 (Figure 7(a) and Table S4). A volcano plot was also used to visualize the changes of DEGs; compared with WT, 207 DEGs were upregulated, including *Hsd3b2*, *Mup16*, *Tfpi2*, *Serpina11*, *Tecta*, and *Serpina3a*, in KO, while 228 DEGs were downregulated, including *Cyp2c38*, *Slc9a1*, *Mroh9*, and *Itch*, in KO (Figure 7(b)). Besides, in Exp2, a total number of 381 DEGs were selected (Figure 7(c) and Table S5); among those, 161 DEGs were upregulated, such as *Kng1* and *Trp63inp1*, while 220 DEGs were downregulated, such as *Tdg-ps*, *Gucy2d*, *Vmn1r185*, and *Ndufa5*, in KO compared with WT (Figure 7(d)). Finally, Figure 7(e) demonstrates that a total number of 45 DEGs were coexpressed in Exp1 and Exp2, including *Tfpi2*, *Tecta*, *Itch*, *Slc9a1*, *Vmn1r185*, and *Enho* (Figure 7(f)).

**Figure 8:**
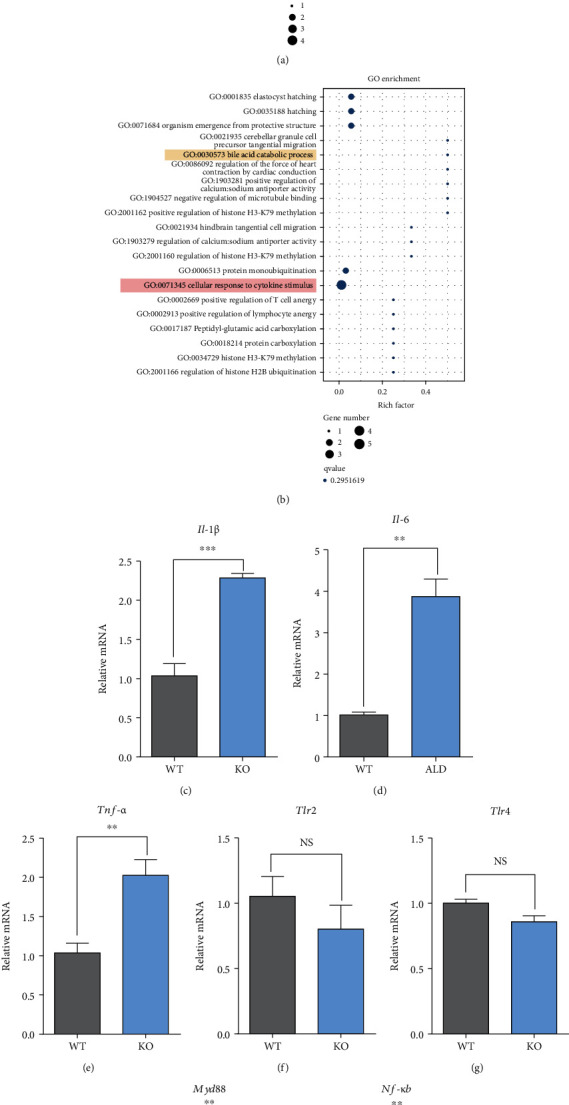
Pathways enrichment results and the relative expression levels of hepatic inflammation pathway-related genes. WT: wild-type (Period2^+/+^) mice; KO: *Per2* gene knockout (Period2^−/−^) mice. (a) KEGG functional analysis of the coexpressed DEGs. (b) Gene ontology (GO) functional analysis of the coexpressed DEGs. The relative expression levels of *Il-1β* (c), *Il-6* (d), *Tnf-α* (e), *Tlr2* (f), *Tlr4* (g), *Myd88* (h), and *Nf-κB* (i) were determined in liver tissues of *Per2* knockout and wild-type mice by RT-PCR method.

To assess the functional consequences, the coexpressed DEGs were further analyzed using the KEGG database and Gene Ontology (GO) database. The enrichment analysis results of KEGG database indicated that KO significantly altered the pathways in inflammation and disease, such as nonalcoholic fatty liver disease, Parkinson disease, Huntington disease, Alzheimer disease, and the TNF signaling pathway (Figure 8(a)). Besides, KO also altered functions in the metabolism, endocrine, and digestive systems, such as primary bile acid biosynthesis, gastric acid secretion, salivary secretion, bile secretion, protein digestion and absorption, steroid hormone biosynthesis, and pancreatic secretion (Figure 8(a)). Next, enrichment analysis using GO database showed that most of DEGs were also enriched in several processes related to metabolism and inflammation functions, such as bile acid catabolic process and cellular response to cytokine stimulus (Figure 8(b)).

### 3.7. Relative Expression Levels of Hepatic Inflammation Pathway-Related Genes

Compared with WT, the relative expression levels of *Il-1β* (Figure 8(c)), *Il-6* (Figure 8(d)), and *Tnf-α* (Figure 8(e)) were significantly increased in KO mice (*P* < 0.01 or *P* < 0.001). However, KO had no impacts on the relative expression levels of *Tlr2* (Figure 8(f)) and *Tlr4* (Figure 8(g)) compared with WT (*P* > 0.05). Finally, the relative expression levels of *Myd88* (Figure 8(h)) and *Nf-κB* (Figure 8(i)) were also upregulated in KO compared with WT (*P* < 0.01).

## 4. Discussion

The key objective of this study was to investigate the mechanisms whereby a *Per2* knockout altered hindgut metabolism and hepatic functions. Our data indicated that *Per2* knockout disturbed the relative expression levels of the circadian rhythm genes in the liver; for example, KO upregulated the relative expression levels of *Per1*, *Per3*, *Cry1*, and *Cry2* while it downregulated the relative expression level of *Bmal1* during ZT4-ZT6; KO also downregulated the relative expression levels of *Per3*, *Cry1*, and *Cry2* while it upregulated the relative expression level of *Clock* during ZT16-ZT18. Russell et al. [37] reported that *Per2* knockout disrupted circadian rhythms and resulted in the imbalance of the hypothalamus-pituitary-adrenal (HPA) axis, thus leading to the augment of the depressive and fright behaviors. Thus, it is concluded that *Per*2 knockout causes a rhythm disorder in the liver of mice. Another report also demonstrated that *Per2* knockout shortened circadian rhythm and reduced mean arterial pressure, heart rate, and exercise frequency in mice [38]. As a consequence, signal transduction in the renin-angiotensin pathway was inhibited and resulted in vascular hypertrophy and abnormal blood pressure [39]. Taken together, data underscored the essentiality of *Per2* for the normal circadian rhythm and physiological functions.

Disturbances of the circadian rhythm will induce imbalance in intestinal microbes and their metabolism. In our study, *Per2* knockout inhibited the TTFLs axis, thus affecting the structure and function of intestinal microbes [9, 10]. We predicted that the alterations of a variety of digestive and metabolic functions in hindgut may result from the disordered gut microbe profiles, because the intestinal microbes can produce key metabolic mediators and further affect the circadian rhythm of the host [40]. Our previous study using 16S rRNA sequencing revealed that KO significantly altered the bacterial genes which enriched in amino acid and carbohydrate metabolism pathways; it is interesting to find that these pathways were consistent with the enrichment results of differentially altered metabolites using metabolomic data in this study. We speculated that *Per2* knockout induced a disorder of amino acid metabolism, and most of intestinal amino acids may enter the citrate cycle pathway and are converted into butyrate to provide energy. The results of differentially altered metabolites in our study were consistent with the hepatic metabolomics data in mice fed with the high-fat diets [41], which suggested that *Per2* knockout may induce an impairment in insulin signaling and lead to increased gluconeogenesis and citrate cycle flux [42], further may contribute to abnormal metabolism and hepatic damage.

The KO also reduced the concentrations of other metabolites which belonge to amino acids, such as GABA, aspartic acid, and glycine. These metabolites can act as the neurotransmitters [43]; for example, GABA is one of the most important inhibitory neurotransmitters in the brain. According to a previous report, the gut microbiota can regulate excitatory and inhibitory neurotransmitters, such as *Lactobacilli* [44]. Thus, the decreased concentrations of these neurotransmitter amino acids in our study may be related to the imbalance of intestinal microbiota. Ono et al. [45] also reported that GABA is necessary to maintain circadian rhythms and stabilize neurotransmitter output signals. Thus, our study further verified the essentiality of GABA to maintain rhythmic oscillations and the key role of the gut microbiota in this process.

Our data also indicated that *Per2* knockout led to increased concentrations of serum glucose and low-density lipoprotein, together with the liver and thymus indexes in mice. These responses may be associated with hepatic dysfunction and excessive fat deposition because of the role of *Per2* in coordinating aspects of lipid metabolism. In agreement with this idea, KO significantly upregulated the relative expression level of *Pparα* but downregulated expression of *Pparγ* compared with WT. KO also upregulated *Cyp7a1* and *Cpt1* compared with WT. Several studies from our laboratory reported that *Per2* silencing downregulated *Pparγ* and suppressed lipid synthesis in bovine mammary epithelial cells [46], underscoring that circadian clock genes control lipid metabolism *via Pparγ* [47, 48]. Several previous studies also showed that *Per2* interacts with key nuclear receptors (*Pparα*, *Pparγ*, and *Rev-erbα*) [49] and plays an important role in the liver transcriptional response to feeding and acute fasting [50]. For example, *Per*2 knockout increased plasma insulin levels due to enhanced glucose-stimulated insulin secretion and impaired insulin clearance in mice [51]. Others also found that mice that lacked a functional Per2 protein in liver exhibit decreased glycogen synthase protein levels during refeeding along with augmented glycogen phosphorylase activity during fasting [50]. The *Per2* mutation mice lack a glucocorticoid rhythm and diurnal feeding rhythm, which will develop obesity when fed a high-fat diet [16, 17]. Several reports also indicated that the synthesis and secretion of bile acids are reduced in *Per2* knockout mice, underscoring a high risk to develop fatty liver [52–54]. Besides, there are relatively few reports on the impacts of *Per2* knockout on the antioxidant capacity. We speculated that dysregulation of circadian rhythms due to the knockout of *Per2* would enhance antioxidant capacity based on the fact that KO increased activities of SOD and GSH-Px but decreased the concentration of MDA.

Rhythm disorders led to an imbalance of intestinal microbiota that affected the adaptability to the environment, diet, immunity, and other functions [55]. Circadian rhythm disturbances also can directly endanger host health and immune function by hindering signal transduction processes in the central circadian clock responsible for production of intestinal metabolites [56]. As such, these alterations ultimately induce derangements in inflammation, metabolism, immunity, and overall health. All these events explain in part the increased incidence of obesity and metabolic diseases among workers who reverse day and night shifts [57] and the physiological effects arising when traveling across time zones [58]. In order to study whether the intestinal metabolic disorder caused by *Per2* knockout impacts metabolism- and inflammation-related functions in the liver, we further screened the differential expressed genes of *Per2* knockout and WT mice of hepatic transcriptome sequencing data from GEO database and verified using RT-PCR. Transcriptome sequencing data indicated that *Per2* knockout altered the expression of several genes in the liver, such as *Tfpi2*, *Slc9a1*, *Enho*, *Pcolce2*, and *Mup-ps1*. The functional prediction results of these genes were highly consistent with our data; for example, KO significantly altered the pathways in hepatic inflammation and disease, such as nonalcoholic fatty liver disease. Besides, KO also altered functions in the metabolism, endocrine, and digestive systems, such as primary bile acid biosynthesis, gastric acid secretion, salivary secretion, bile secretion, protein digestion and absorption, steroid hormone biosynthesis, and pancreatic secretion. It is also worth noting that several genes also enriched in the TNF signaling pathway and cellular response to cytokine stimulus from KEGG and GO databases, which reflected that KO may induce hepatic inflammation responses. We also detected alterations of inflammatory-related factors, and data indicated that compared with WT, the relative expression levels of *Il-1β*, *Il-6*, and *Tnf-α*, as well as *Myd88* and *Nf-κB*, were upregulated in KO mice, which suggested that KO may induce hepatic inflammation through the Tlr signaling and *Nf-κB* pathways [59].

In summary, our data are consistent with the hypothesis; *Per2* knockout altered intestinal amino acids and carbohydrate metabolism; for example, KO decreased the concentrations of amino acids such as GABA, aspartic acid, glycine, L-allothreonine, methionine, proline, serine, and valine while increased the concentrations of carbohydrates such as cellobiose, D-talose, fucose, lyxose, and xylose. Moreover, the imbalance of intestinal metabolism further may induce liver dysfunctions. Firstly, KO induced hepatic lipid metabolism disordered, because of the increase of liver index and serum concentrations of low-density lipoprotein, and upregulated lipid metabolism-related genes, such as *Pparα*, *Cyp7a1*, and *Cpt1*. Then, KO also improved hepatic antioxidant capacity due to the increased activities of SOD and GSH-Px and the decreased concentration of MDA. Moreover, KO increased the relative expression levels of hepatic inflammation-related genes, such as *Il-1β*, *Il-6*, *Tnf-α*, *Myd88*, and *Nf-κB p65*, which may potentially lead to hepatic inflammation.

## Data Availability

The original data presented in this study are included in the article and supplementary material; further inquiries can be directed to the corresponding author.

## Supplementary Materials

Supplementary Materials Table S1: specific primers used for RT-PCR. Table S2: the significantly differential metabolites in metabolomics data in KO and WT mice. Table S3: KEGG pathways enriched with significantly altered metabolites in KO and WT mice. Table S4: identification of DEGs in WT and KO mice in Exp1. Table S5: identification of DEGs in WT and KO mice in Exp2.
