# Supplementary material for: Impacts of Circadian Gene Period2 Knockout on Intestinal Metabolism and Hepatic Antioxidant and Inflammation State in Mice
Source: Oxid Med Cell Longev. 2022 Jul 19;2022:7896371. doi: 10.1155/2022/7896371 (PMC9325607; doi:10.1155/2022/7896371)
Supplement: Supplementary Materials — Table S1: specific primers used for RT-PCR. Table S2: the significantly differential metabolites in metabolomics data in KO and WT mice. Table S3: KEGG pathways enriched with significantly altered metabolites in KO and WT mice. Table S4: identification of DEGs in WT and KO mice in Exp1. Table S5: identification of DEGs in WT and KO mice in Exp2. [file 7896371.f1.zip › Supplementary data (1).docx]

**Impacts of Circadian Gene Per2 Knockout on Intestinal Metabolism and Hepatic Antioxidant and Inflammation in Mice**

**Supplementary Materials**

**Table S1. Specific primers used for RT-PCR.**

| Name | Primer Name | Sequence (5’-3’) | GenBank accession |
| --- | --- | --- | --- |
| *Per1* | *Per1*-F | GAAACGGCAAGCGGATGGA | NM_011065.5 |
|  | *Per1*-R | GGAAGGTGAAGAAGCCACCA |  |
| *Per3* | *Per3*-F | AAAAGATCCTGACCTCGCCC | NM_001289877.1 |
|  | *Per3*-R | AGTGGACCCTGCTTGAACAC |  |
| *Bmal1* | *Bmal1*-F | CCACCTCAGAGCCATTGATACA | XM_030242022.2 |
|  | *Bmal1*-R | GAGCAGGTTTAGTTCCACTTTGTCT |  |
| *Cry1* | *Cry1*-F | CACTGGTTCCGAAAGGGACTC | NM_007771.3 |
|  | *Cry1*-R | CTGAAGCAAAAATCGCCACCT |  |
| *Cry2* | *Cry2*-F | GGTTCCGCAAAGGACTACGG | NM_009963.4 |
|  | *Cry2*-R | ATCGGTTGATGCCCACAGAC |  |
| *Pparα* | *Pparα*-F | CCTCAGGGTACCACTACGGAGT | XM_030248424.2 |
|  | *Pparα*-R | GCCGAATAGTTCGCCGAA |  |
| *Pparγ* | *Pparγ*-F | AGGCCGAGAAGGAGAAGCTGTTG | XM_036165927.1 |
|  | *Pparγ*-R | TGGCCACCTCTTTGCTGTGCTC |  |
| *Cyp7a1* | *Cyp7a1*-F | AGCAACTAAACAACCTGCCAGTACTA | NM_007824.3 |
|  | *Cyp7a1*-R | GTCCGGATATTCAAGGATGCA |  |
| *Cpt1* | *Cpt1*-F | AAAGATCAATCGGACCCTAGACA | NM_013495.2 |
|  | *Cpt1*-R | CAGCGAGTAGCGCATAGTCA |  |
| *Il-1β* | *Il-1β*-F | TGCCACCTTTTGACAGTGATG | NM_008361.4 |
|  | *Il-1β*-R | ATGTGCTGCTGCGAGATTTG |  |
| *Il-6* | *Il-6*-F | TGATGGATGCTACCAAACTGGA | NM_001314054.1 |
|  | *Il-6*-R | GTGACTCCAGCTTATCTCTTGGT |  |
| *Tlr4* | *Tlr4*-F | TCTGGGGAGGCACATCTTCT | NM_021297.3 |
|  | *Tlr4*-R | AGGTCCAAGTTGCCGTTTCT |  |
| *Tlr2* | *Tlr2*-F | ACCCGCCCTTTAAGCTGTGT | NM_011905.3 |
|  | *Tlr2*-R | TCGTACTTGCACCACTCGCT |  |
| *Myd88* | *Myd88*-F | ACTGGCCTGAGCAACTAGGA | NM_010851.3 |
|  | *Myd88*-R | CGTGCCACTACCTGTAGCAA |  |
| *Nf-κb* | *Nf-κb*-F | GAGGCACGAGGCTCCTTTTCT | XM_006531695.3 |
|  | *Nf-κb*-R | GTAGCTGCATGGAGACTCGAACA |  |
| *Gapdh* | *Gapdh* -F | AGGTCGGTGTGAACGGATTTG | NM_001289726.1 |
|  | *Gapdh* -R | TGTAGACCATGTAGTTGAGGTCA |  |

**Table S2.** **The significantly differential metabolites in Metabolomics data in KO and WT mice**

| Metabolites | Mean (KO) | Mean (CON) | VIP | *P*-value | FC |
| --- | --- | --- | --- | --- | --- |
| Analyte 11 | 0.009 | 0.006 | 1.67 | 0.01 | 1.54 |
| Analyte 1207 | 0.001 | 0.010 | 1.46 | 0.04 | 0.14 |
| Analyte 128 | 0.026 | 0.014 | 1.55 | 0.05 | 1.86 |
| Analyte 5 | 0.299 | 0.200 | 1.71 | 0.01 | 1.49 |
| Analyte 6 | 0.016 | 0.004 | 1.59 | 0.02 | 4.60 |
| Analyte 198 | 0.045 | 0.020 | 1.29 | 0.04 | 2.26 |
| Analyte 1026 | 0.004 | 0.001 | 1.74 | 0.04 | 7.00 |
| Analyte 581 | 0.006 | 0.003 | 1.73 | 0.02 | 1.82 |
| Ureidosuccinic acid | 0.005 | 0.002 | 1.88 | 0.02 | 2.94 |
| Unknown | 0.005 | 0.001 | 1.10 | 0.03 | 4.33 |
| Glutathione | 0.003 | 0.000 | 2.03 | 0.01 | 10.33 |
| Xanthosine | 0.002 | 0.000 | 1.61 | 0.03 | 9.50 |
| Naringenin | 0.036 | 0.010 | 1.69 | 0.05 | 3.66 |
| Analyte 1329 | 0.008 | 0.001 | 2.06 | 0.00 | 7.50 |
| Analyte 383 | 0.003 | 0.001 | 1.87 | 0.01 | 1.86 |
| Analyte 155 | 0.009 | 0.006 | 2.10 | 0.00 | 1.52 |
| Unknown | 0.000 | 0.030 | 1.01 | 0.03 | 0.00 |
| Unknown | 0.022 | 0.000 | 1.45 | 0.02 | —— |
| Analyte 1324 | 0.004 | 0.000 | 1.53 | 0.02 | 18.00 |
| Analyte 1042 | 0.016 | 0.007 | 1.61 | 0.03 | 2.25 |
| Analyte 1353 | 0.001 | 0.012 | 1.74 | 0.02 | 0.11 |
| Corticosterone | 1.220 | 0.437 | 1.68 | 0.04 | 2.79 |
| 21-Hydroxypregnenolone | 0.033 | 0.262 | 1.70 | 0.01 | 0.13 |
| Glucose-1-phosphate | 0.058 | 0.031 | 1.85 | 0.02 | 1.89 |
| Coprostan-3-one | 0.011 | 0.086 | 1.49 | 0.05 | 0.12 |
| Indoleacetic acid | 0.004 | 0.042 | 1.48 | 0.05 | 0.09 |
| Succinic acid semialdehyde | 0.000 | 0.002 | 1.55 | 0.04 | 0.09 |
| 5-(2-Hydroxyethyl)-4-methylthiazole | 0.025 | 0.006 | 1.99 | 0.01 | 4.32 |
| 24,25-Dihydrolanosterol | 2.663 | 1.049 | 1.78 | 0.04 | 2.54 |
| 2-Monopalmitin | 0.008 | 0.002 | 1.67 | 0.03 | 4.88 |
| Aminomalonic acid | 0.004 | 0.025 | 2.05 | 0.00 | 0.17 |
| GABA | 0.025 | 0.056 | 1.54 | 0.04 | 0.45 |
| Pentadecanoic acid | 0.178 | 0.340 | 1.61 | 0.03 | 0.52 |
| Azelaic acid | 0.100 | 0.061 | 1.65 | 0.04 | 1.65 |
| Methionine | 0.134 | 0.384 | 1.65 | 0.03 | 0.35 |
| Fucose | 0.282 | 0.134 | 1.73 | 0.01 | 2.11 |
| 3-Hydroxybutyric acid | 0.094 | 0.033 | 1.46 | 0.03 | 2.83 |
| 2-Hydroxybutanoic acid | 0.010 | 0.014 | 1.56 | 0.05 | 0.70 |
| Fumaric acid | 0.016 | 0.081 | 1.90 | 0.01 | 0.19 |
| Aspartic acid | 0.035 | 0.563 | 1.69 | 0.03 | 0.06 |
| Proline | 1.166 | 2.366 | 1.65 | 0.03 | 0.49 |
| Cellobiose | 0.214 | 0.067 | 1.67 | 0.02 | 3.18 |
| D-Talose | 0.983 | 0.592 | 1.61 | 0.03 | 1.66 |
| Serine | 0.234 | 0.632 | 2.04 | 0.01 | 0.37 |
| N-Methyl-DL-alanine | 0.094 | 0.255 | 1.96 | 0.01 | 0.37 |
| L-Allothreonine | 0.124 | 0.329 | 1.67 | 0.03 | 0.38 |
| Lyxose | 0.975 | 0.341 | 2.01 | 0.01 | 2.86 |
| Xylose | 11.885 | 4.979 | 1.83 | 0.01 | 2.39 |
| Glycine | 0.439 | 1.349 | 1.91 | 0.01 | 0.33 |
| Valine | 0.914 | 2.218 | 1.60 | 0.04 | 0.41 |

WT, wild-type (*Period2^+/+^*) mice; KO, *Per2* gene knockout (*Period2^-/-^*) mice; VIP, variable importance in projection; FC, fold change; Analyte plus Number represented unrecognized metabolites and unknown represented unknown metabolites.

**Table S3. KEGG pathways enriched with significantly altered metabolites in KO and WT mice**

| **Metabolic pathway** | **Significantly altered metabolites** | **Trends** |
| --- | --- | --- |
| Alanine, aspartate and glutamate metabolism | (0.061) Aspartic acid | down |
|  | (0.087) Succinate semialdehyde | down |
|  | (0.451) GABA | down |
|  | (0.195) Fumaric acid | down |
|  | (2.944) Carbamoyl-aspartic acid | up |
| Aminoacyl-tRNA biosynthesis | (0.325) Glycine | down |
|  | (0.061) Aspartic acid | down |
|  | (0.371) Serine | down |
|  | (0.349) Methionine | down |
|  | (0.412) Valine | down |
|  | (0.493) Proline | down |
| Butanoate metabolism | (2.826) 3-Hydroxybutyric acid | up |
|  | (0.451) GABA | down |
|  | (0.087) Succinate semialdehyde | down |
| Glycine, serine and threonine metabolism | (0.371) Serine | down |
|  | (0.325) Glycine | down |
|  | (0.377) L-Allothreonine | down |
| Glyoxylate and dicarboxylate metabolism | (0.371) Serine | down |
|  | (0.325) Glycine | down |
| Glutathione metabolism | (10.333) Glutathione | up |
|  | (0.325) Glycine | down |
| Cysteine and methionine metabolism | (0.371) Serine | down |
|  | (0.349) Methionine | down |
| Arginine and proline metabolism | (0.451) GABA | down |
|  | (0.493) Proline | down |

The number in the parentheses represents the value of fold change (FC). Up and down indicate that the metabolites were up-regulated and down-regulated of KO and CON mice under the LD cycles of 12L:12D and 8L:16D.
