# Supplementary material for: Impacts of Circadian Gene Period2 Knockout on Intestinal Metabolism and Hepatic Antioxidant and Inflammation State in Mice
Source: Oxid Med Cell Longev. 2022 Jul 19;2022:7896371. doi: 10.1155/2022/7896371 (PMC9325607; doi:10.1155/2022/7896371)
Supplement: Supplementary Materials — Table S1: specific primers used for RT-PCR. Table S2: the significantly differential metabolites in metabolomics data in KO and WT mice. Table S3: KEGG pathways enriched with significantly altered metabolites in KO and WT mice. Table S4: identification of DEGs in WT and KO mice in Exp1. Table S5: identification of DEGs in WT and KO mice in Exp2. [file 7896371.f1.zip › Table S4 Identification of DEGs in WT and KO mice in Exp1.docx]

Table S4. Identification of DEGs in WT and KO mice in Exp1

| Gene id | KO1 | KO2 | KO3 | WT1 | WT2 | WT3 | log2FoldChange | *P*-value |
| --- | --- | --- | --- | --- | --- | --- | --- | --- |
| *Hsd3b2* | 1800.17 | 1256.15 | 1743.09 | 5.72951 | 12.0875 | 1.37304 | 7.986006476 | 1.2E-73 |
| *Cyp2c38* | 5.03309 | 12.8679 | 18.835 | 385.787 | 448.045 | 434.567 | -5.00147382 | 2.4E-51 |
| *Mup16* | 6095.07 | 1818.66 | 5365.41 | 0 | 0 | 0 | 14.52799767 | 4.5E-25 |
| *Itgad* | 1276.73 | 1107.86 | 1279.92 | 444.992 | 472.22 | 399.555 | 1.476775174 | 3.8E-23 |
| *Tfpi2* | 724.765 | 627.462 | 689.19 | 240.639 | 250.615 | 243.028 | 1.470162402 | 4.6E-22 |
| *Serpina11* | 31423.3 | 16179.2 | 32451 | 3722.27 | 5126.73 | 5223.04 | 2.507607076 | 1.5E-19 |
| *Serpina3a* | 298.63 | 198.533 | 101.024 | 0 | 5.64085 | 1.37304 | 6.201750648 | 2.5E-17 |
| *Tecta* | 1848.82 | 1652.6 | 1808.16 | 563.402 | 536.686 | 755.172 | 1.512316928 | 2.8E-17 |
| *Itch* | 694.566 | 785.553 | 799.632 | 2100.82 | 3202.39 | 2123.41 | -1.703215004 | 2.4E-16 |
| *Slc25a22* | 3373.85 | 3702.88 | 3433.11 | 1552.7 | 1875.98 | 1748.57 | 1.019371422 | 4.2E-16 |
| *Slc9a1* | 67.1079 | 72.3052 | 51.3682 | 255.918 | 487.53 | 389.257 | -2.576932988 | 4.3E-16 |
| *Obox3-ps1* | 303.663 | 307.603 | 422.932 | 40.1066 | 72.5252 | 75.5172 | 2.421480201 | 1.9E-15 |
| *Mroh9* | 31.8762 | 52.697 | 47.9437 | 273.107 | 195.012 | 319.918 | -2.540079212 | 6.4E-15 |
| *Vmn1r185* | 271.787 | 342.53 | 212.322 | 891.894 | 984.731 | 1363.43 | -1.970231747 | 1.1E-14 |
| *Gucy2e* | 45.2978 | 30.6378 | 36.8139 | 211.992 | 351.344 | 186.733 | -2.761875546 | 2.5E-14 |
| *Gm28035* | 0 | 0 | 0 | 150.877 | 84.6127 | 96.7993 | -9.283898011 | 2E-13 |
| *Sigmar1* | 1365.64 | 1193.65 | 1277.36 | 576.771 | 631.775 | 683.774 | 1.01236862 | 2.9E-13 |
| *Eldr* | 93.951 | 77.82 | 118.147 | 0 | 3.22334 | 0 | 6.289652302 | 5.9E-13 |
| *Irf5* | 312.052 | 215.69 | 184.926 | 57.2951 | 36.2626 | 29.5204 | 2.57663283 | 1.4E-12 |
| *Phyhd1* | 100.662 | 75.9817 | 89.8944 | 0 | 0 | 0 | 8.883793531 | 1.3E-12 |
| *Mup18* | 471.433 | 268.387 | 511.114 | 36.2869 | 85.4186 | 35.699 | 2.970422384 | 1.9E-12 |
| *Gm7953* | 350.639 | 408.095 | 381.837 | 169.975 | 136.186 | 128.379 | 1.415668464 | 3.2E-12 |
| *Cmtm2b* | 2006.52 | 2469.41 | 1533.34 | 145.148 | 485.113 | 187.42 | 2.871079191 | 3.7E-12 |
| *Trit1* | 0 | 15.3189 | 5.99296 | 217.721 | 248.197 | 103.665 | -4.585026959 | 1.3E-11 |
| *Enho* | 543.574 | 259.808 | 531.661 | 76.3935 | 103.953 | 91.3072 | 2.283276276 | 1.6E-11 |
| *Fyb2* | 73.8186 | 56.3735 | 74.4839 | 0 | 0 | 0 | 8.504061633 | 2.4E-11 |
| *Nfix* | 520.086 | 476.724 | 452.04 | 926.271 | 1393.29 | 1240.54 | -1.302948634 | 2.7E-11 |
| *Cyp4a31* | 109.05 | 63.7266 | 46.2314 | 0 | 0 | 0 | 8.59486588 | 6.4E-11 |
| *Tmem254* | 494.92 | 414.836 | 425.5 | 137.508 | 198.236 | 172.317 | 1.374293738 | 6.7E-11 |
| *Ms4a8a* | 57.0417 | 52.697 | 51.3682 | 0 | 0 | 0 | 8.160767198 | 2.3E-10 |
| *Gm6614* | 88.9179 | 63.7266 | 115.578 | 5.72951 | 12.8934 | 8.92476 | 3.213138164 | 3E-10 |
| *Bet1* | 860.658 | 816.803 | 916.922 | 303.664 | 419.034 | 429.762 | 1.15894868 | 3.4E-10 |
| *Gm30784* | 35.2316 | 36.1526 | 48.7998 | 135.598 | 146.662 | 174.376 | -1.924110643 | 3.4E-10 |
| *Cyp2c68* | 0 | 3.06378 | 0 | 53.4754 | 60.4377 | 87.188 | -5.635857307 | 4.1E-10 |
| *Mrps18a* | 52.0086 | 56.9863 | 44.5191 | 0 | 0 | 0 | 8.094167542 | 4.4E-10 |
| *Peg3* | 33.5539 | 36.1526 | 46.2314 | 150.877 | 134.575 | 133.871 | -1.836894971 | 5.9E-10 |
| *Vkorc1l1* | 1380.74 | 1413.63 | 1467.42 | 3029 | 5818.94 | 3528.71 | -1.538648665 | 6.3E-10 |
| *Gm7631* | 2372.26 | 2944.9 | 3819.23 | 1264.31 | 1384.43 | 1293.4 | 1.213114698 | 8.2E-10 |
| *Stra6l* | 1914.25 | 1810.69 | 1708.85 | 637.885 | 937.992 | 904.147 | 1.125030385 | 8.6E-10 |
| *Anks3* | 115.761 | 120.1 | 124.14 | 338.041 | 431.122 | 271.862 | -1.529690354 | 1E-09 |
| *Gm11340* | 78.8517 | 59.4373 | 104.449 | 3.81967 | 9.67003 | 10.2978 | 3.24372607 | 9.8E-10 |
| *C6* | 52.0086 | 31.2505 | 68.4909 | 0 | 0 | 0 | 8.073105795 | 1.8E-09 |
| *Gm15046* | 85.5625 | 55.7608 | 65.9225 | 9.54918 | 10.4759 | 6.17868 | 3.00110461 | 4.2E-09 |
| *Cutal* | 870.724 | 743.885 | 535.085 | 259.738 | 266.732 | 312.367 | 1.350833869 | 5.3E-09 |
| *Gusb* | 567.061 | 515.94 | 604.433 | 977.836 | 1548.01 | 1551.54 | -1.27683471 | 5.5E-09 |
| *Gm10054* | 248.299 | 239.587 | 232.869 | 99.3115 | 94.2827 | 63.8464 | 1.504727543 | 7.2E-09 |
| *Gm15161* | 97.3064 | 53.3097 | 83.0453 | 11.459 | 12.0875 | 9.61128 | 2.814741061 | 8.2E-09 |
| *Eif4b* | 662.69 | 655.036 | 721.723 | 234.91 | 229.663 | 359.737 | 1.298514329 | 1.3E-08 |
| *Cyp4f15* | 461.366 | 506.136 | 418.651 | 150.877 | 232.886 | 159.273 | 1.342899895 | 1.5E-08 |
| *Gm43123* | 154.348 | 158.091 | 130.989 | 320.853 | 526.211 | 364.542 | -1.4564996 | 2E-08 |
| *4930588K23Rik* | 114.083 | 0 | 0 | 0 | 0 | 0 | 21.88234702 | 2.2E-08 |
| *Nup85* | 35.2316 | 40.4419 | 30.8209 | 120.32 | 114.429 | 116.708 | -1.712497384 | 2.3E-08 |
| *Fcamr* | 0 | 0 | 0 | 22.918 | 38.6801 | 45.3103 | -7.673843036 | 2.5E-08 |
| *Gm42654* | 145.96 | 77.82 | 95.0312 | 383.877 | 493.171 | 271.175 | -1.867559112 | 3E-08 |
| *Aox3* | 553.64 | 498.17 | 601.864 | 64.9344 | 146.662 | 198.404 | 1.989694623 | 3.2E-08 |
| *Zfp786* | 1399.2 | 1413.63 | 953.736 | 427.803 | 618.076 | 527.247 | 1.254158376 | 3.4E-08 |
| *Gm6309* | 129.183 | 109.683 | 95.0312 | 689.451 | 247.391 | 473.699 | -2.078850671 | 3.5E-08 |
| *D030068K23Rik* | 4496.23 | 2935.71 | 4660.81 | 7960.2 | 12944.9 | 9874.9 | -1.348562303 | 3.5E-08 |
| *Tspan33* | 129.183 | 98.0409 | 123.284 | 26.7377 | 37.8743 | 30.8934 | 1.845464647 | 4.1E-08 |
| *Gm14325* | 52.0086 | 54.5353 | 50.5121 | 148.967 | 129.74 | 158.586 | -1.473929648 | 4.9E-08 |
| *Nopchap1* | 499.953 | 248.779 | 405.809 | 129.869 | 83.8069 | 120.141 | 1.793409658 | 5.5E-08 |
| *Coro1a* | 28.5208 | 34.3143 | 31.6771 | 0 | 0 | 0 | 7.397901055 | 5.5E-08 |
| *Ncald* | 204.679 | 146.449 | 184.069 | 45.8361 | 69.3018 | 43.2508 | 1.738625001 | 7.1E-08 |
| *Man1b1* | 218.101 | 235.911 | 188.35 | 95.4918 | 71.7194 | 88.5611 | 1.342338692 | 7.2E-08 |
| *Mup-ps2* | 181.191 | 11.6424 | 24.828 | 0 | 0 | 0 | 8.58633987 | 1E-07 |
| *Gm8702* | 26.8431 | 26.9613 | 31.6771 | 131.779 | 113.623 | 83.0689 | -1.924894815 | 1E-07 |
| *Tox* | 28.5208 | 58.2118 | 29.9648 | 147.057 | 152.303 | 177.809 | -1.999051213 | 1.1E-07 |
| *Pde4dip* | 409.358 | 313.118 | 247.424 | 683.721 | 1087.88 | 773.022 | -1.400191785 | 1.4E-07 |
| *Dgcr8* | 327.151 | 346.207 | 321.907 | 674.172 | 1124.95 | 667.984 | -1.311261941 | 1.5E-07 |
| *Rnf220* | 142.604 | 104.781 | 190.918 | 704.73 | 520.57 | 326.784 | -1.821331292 | 1.9E-07 |
| *Yod1* | 458.011 | 387.874 | 320.195 | 105.041 | 131.351 | 183.987 | 1.451226223 | 2E-07 |
| *Egfr* | 273.464 | 167.895 | 272.251 | 24.8279 | 41.9034 | 77.5768 | 2.26462353 | 2E-07 |
| *Atp8b2* | 112.406 | 106.007 | 140.406 | 231.09 | 365.849 | 396.809 | -1.477571507 | 2.1E-07 |
| *Atp12a* | 31.8762 | 26.3485 | 32.5332 | 74.4836 | 232.886 | 173.003 | -2.42313579 | 2.3E-07 |
| *Als2* | 0 | 0 | 0 | 22.918 | 20.1459 | 39.8182 | -7.300447263 | 3.2E-07 |
| *Serpina12* | 25.1654 | 36.1526 | 20.5473 | 0 | 0 | 0 | 7.191967654 | 3.6E-07 |
| *Clip1* | 0 | 0 | 0 | 32.4672 | 19.3401 | 24.7147 | -7.165109718 | 4.5E-07 |
| *Slc25a51* | 640.88 | 517.779 | 672.067 | 1119.16 | 1489.18 | 1046.26 | -1.000317644 | 8.3E-07 |
| *Def6* | 0 | 0 | 0 | 22.918 | 23.3692 | 21.2821 | -6.996312545 | 9.6E-07 |
| *Cdc14b* | 541.896 | 723.665 | 534.229 | 1396.09 | 1680.17 | 998.887 | -1.176041507 | 1.1E-06 |
| *5830437K03Rik* | 466.4 | 402.581 | 338.174 | 215.812 | 182.925 | 202.523 | 1.006192176 | 1.1E-06 |
| *Gm12617* | 8.38848 | 12.8679 | 9.4175 | 38.1967 | 70.1077 | 111.903 | -2.829854563 | 1.1E-06 |
| *Crtc1* | 162.737 | 194.244 | 130.989 | 297.934 | 564.891 | 450.357 | -1.432126335 | 1.6E-06 |
| *Gbp4* | 43.6201 | 41.6674 | 45.3752 | 7.63935 | 8.05835 | 4.80564 | 2.707478309 | 1.7E-06 |
| *Zyg11b* | 4917.33 | 3150.18 | 5125.69 | 1147.81 | 1618.92 | 2308.08 | 1.376188463 | 1.8E-06 |
| *Gm35572* | 104.017 | 88.8496 | 85.6137 | 9.54918 | 24.1751 | 4.80564 | 2.831150648 | 2.4E-06 |
| *Lcat* | 159.381 | 115.198 | 130.989 | 55.3853 | 45.9326 | 52.862 | 1.393609957 | 2.6E-06 |
| *Tcf7l1* | 471.433 | 655.036 | 541.935 | 1143.99 | 1014.55 | 1882.44 | -1.274986829 | 2.6E-06 |
| *Acy3* | 837.17 | 396.453 | 967.435 | 299.844 | 208.711 | 179.868 | 1.683194066 | 2.7E-06 |
| *Aldh1a7* | 147.637 | 187.503 | 172.083 | 61.1148 | 58.826 | 82.3824 | 1.318899876 | 3E-06 |
| *Obox7* | 23.4877 | 13.4806 | 27.3964 | 0 | 0 | 0 | 6.83304285 | 3.8E-06 |
| *Gm31305* | 15.0993 | 11.6424 | 9.4175 | 45.8361 | 53.1851 | 109.843 | -2.575094475 | 4.4E-06 |
| *Olfr477* | 48.6532 | 294.123 | 209.754 | 26.7377 | 30.6217 | 26.0878 | 2.733148549 | 4.6E-06 |
| *Retreg1* | 672.756 | 684.448 | 1063.32 | 1485.85 | 1586.69 | 1815.16 | -1.013150778 | 5E-06 |
| *Adcy7* | 23.4877 | 23.2847 | 22.2596 | 61.1148 | 78.166 | 109.843 | -1.870066465 | 5.8E-06 |
| *Gm47255* | 8.38848 | 9.19134 | 5.99296 | 42.0164 | 36.2626 | 67.279 | -2.634670694 | 6E-06 |
| *Slc15a4* | 20.1324 | 18.9954 | 30.8209 | 93.582 | 96.7003 | 66.5924 | -1.863696119 | 6.5E-06 |
| *Gm37163* | 872.402 | 716.311 | 773.948 | 229.18 | 258.673 | 464.088 | 1.30161435 | 6.9E-06 |
| *4931431B13Rik* | 256.688 | 166.057 | 220.027 | 57.2951 | 103.147 | 60.4138 | 1.52042073 | 9E-06 |
| *Aox1* | 145.96 | 139.096 | 217.459 | 42.0164 | 43.5151 | 74.1442 | 1.633646219 | 1E-05 |
| *Gm18726* | 0 | 0 | 0.85614 | 17.1885 | 22.5634 | 32.953 | -6.147922741 | 1.1E-05 |
| *Camkk2* | 38.587 | 32.4761 | 30.8209 | 87.8525 | 90.2536 | 91.3072 | -1.423193413 | 1.1E-05 |
| *Slc36a1* | 8457.27 | 3694.3 | 10790.7 | 1080.97 | 1880.82 | 543.037 | 2.710518033 | 1.3E-05 |
| *H1f3* | 582.161 | 526.97 | 272.251 | 84.0328 | 185.342 | 51.489 | 2.100578175 | 1.3E-05 |
| *Esp16* | 194.613 | 572.314 | 448.616 | 150.877 | 99.1178 | 59.7272 | 1.993412738 | 1.4E-05 |
| *Rbm15* | 28.5208 | 16.5444 | 11.1298 | 0 | 0 | 0 | 6.627632593 | 1.6E-05 |
| *Gm47204* | 312.052 | 364.59 | 148.112 | 28.6475 | 78.166 | 88.5611 | 2.050612256 | 1.8E-05 |
| *Filip1l* | 2030.01 | 5952.92 | 4213.05 | 1669.2 | 1184.58 | 580.796 | 1.830465743 | 1.9E-05 |
| *Mup-ps3* | 278.498 | 34.9271 | 643.815 | 0 | 4.02918 | 0 | 7.795714671 | 2.2E-05 |
| *Dnai1* | 21.8101 | 11.6424 | 17.1227 | 0 | 0 | 0 | 6.479064533 | 2.3E-05 |
| *Gm10505* | 50.3309 | 58.2118 | 65.0664 | 147.057 | 132.963 | 118.768 | -1.178198204 | 2.4E-05 |
| *Cdh5* | 149.315 | 101.105 | 95.0312 | 28.6475 | 21.7576 | 46.6834 | 1.804057662 | 2.6E-05 |
| *Gm5565* | 80.5294 | 65.5649 | 75.34 | 303.664 | 131.351 | 205.269 | -1.526865975 | 2.7E-05 |
| *Col7a1* | 78.8517 | 83.9475 | 129.277 | 173.795 | 278.819 | 304.815 | -1.377064578 | 3.4E-05 |
| *Gm6185* | 21.8101 | 32.4761 | 33.3893 | 76.3935 | 82.1952 | 94.0532 | -1.502353155 | 3.5E-05 |
| *Pfkfb2* | 73.8186 | 65.5649 | 58.2173 | 13.3689 | 20.9517 | 22.6552 | 1.73800192 | 3.5E-05 |
| *Trpm1* | 20.1324 | 28.7995 | 12.8421 | 55.3853 | 92.6711 | 100.232 | -2.005458101 | 3.7E-05 |
| *Zbtb16* | 0 | 0 | 0 | 13.3689 | 18.5342 | 12.3574 | -6.390286162 | 4.3E-05 |
| *Rtl10* | 0 | 0 | 0 | 15.2787 | 16.1167 | 12.3574 | -6.367469252 | 4.3E-05 |
| *Lrrc8a* | 31.8762 | 36.1526 | 36.8139 | 1.90984 | 8.05835 | 2.74608 | 2.956413245 | 4.6E-05 |
| *Lingo3* | 166.092 | 120.713 | 189.206 | 278.836 | 344.898 | 529.993 | -1.287338864 | 4.9E-05 |
| *Zfp672* | 820.393 | 699.154 | 630.117 | 185.254 | 238.527 | 437.313 | 1.309060499 | 5.3E-05 |
| *Cyp3a13* | 50.3309 | 66.1776 | 66.7787 | 114.59 | 149.08 | 150.348 | -1.168934121 | 5.3E-05 |
| *Zfp991* | 20.1324 | 28.7995 | 11.9859 | 64.9344 | 130.545 | 67.9655 | -2.106995852 | 5.9E-05 |
| *C9* | 16.777 | 12.8679 | 12.8421 | 0 | 0 | 0 | 6.232949267 | 6.7E-05 |
| *Alg2* | 176.158 | 348.658 | 237.15 | 110.771 | 88.6419 | 115.335 | 1.287031657 | 6.7E-05 |
| *Nlrp4a* | 0 | 1.83827 | 0 | 26.7377 | 12.0875 | 41.8777 | -5.081259261 | 6.8E-05 |
| *Pcolce2* | 68.7855 | 39.2164 | 77.9084 | 11.459 | 7.25252 | 18.536 | 2.292437532 | 7E-05 |
| *Cp* | 0 | 0 | 0 | 15.2787 | 11.2817 | 14.4169 | -6.270871571 | 7.2E-05 |
| *Cecr2* | 0 | 0 | 0 | 17.1885 | 8.86419 | 15.1034 | -6.272011302 | 9.2E-05 |
| *Hsd3b6* | 187.902 | 177.699 | 196.055 | 59.2049 | 107.982 | 85.1285 | 1.12736307 | 9.1E-05 |
| *Gm867* | 58.7194 | 53.3097 | 52.2243 | 122.23 | 125.71 | 103.665 | -1.101882121 | 9.3E-05 |
| *Gm6482* | 0 | 0 | 0 | 24.8279 | 9.67003 | 9.61128 | -6.354712396 | 9.8E-05 |
| *Zscan30* | 0 | 0 | 0 | 11.459 | 15.3109 | 12.3574 | -6.214297198 | 0.0001 |
| *Gm43309* | 2243.08 | 2018.42 | 2023.05 | 401.066 | 1022.61 | 1114.91 | 1.303348343 | 0.00011 |
| *Tmc7* | 57.0417 | 67.4031 | 57.3612 | 143.238 | 108.788 | 149.661 | -1.131223672 | 0.00011 |
| *n-R5s13* | 45.2978 | 23.2847 | 27.3964 | 5.72951 | 2.41751 | 5.49216 | 2.837728265 | 0.00011 |
| *Obi1* | 177.836 | 825.382 | 506.833 | 143.238 | 166.808 | 82.3824 | 1.950636275 | 0.00011 |
| *Ube2v1* | 1.6777 | 0 | 0 | 28.6475 | 11.2817 | 16.4765 | -5.740483546 | 0.00012 |
| *Gm49588* | 187.902 | 195.469 | 169.515 | 330.402 | 633.387 | 317.859 | -1.21636583 | 0.00012 |
| *Ptpn5* | 1548.51 | 1409.95 | 1299.62 | 437.353 | 942.022 | 695.445 | 1.03156269 | 0.00015 |
| *Sh3bp5l* | 216.423 | 237.136 | 234.581 | 63.0246 | 112.011 | 131.125 | 1.141872811 | 0.00018 |
| *Gm16399* | 16.777 | 43.5057 | 17.1227 | 0 | 2.41751 | 2.74608 | 3.703594457 | 0.00019 |
| *Ginm1* | 0 | 0.61276 | 0 | 1.90984 | 29.0101 | 43.2508 | -6.207239505 | 0.0002 |
| *Dop1b* | 0 | 0 | 0 | 11.459 | 11.2817 | 11.6708 | -6.024079472 | 0.00024 |
| *Gm8130* | 114.083 | 111.522 | 89.8944 | 28.6475 | 41.9034 | 53.5486 | 1.304900245 | 0.00026 |
| *Gm47862* | 82.2071 | 160.542 | 172.083 | 261.648 | 301.382 | 484.683 | -1.328594452 | 0.00027 |
| *Gm15567* | 41.9424 | 67.4031 | 19.6911 | 116.5 | 169.225 | 143.483 | -1.727288515 | 0.00027 |
| *Ifi47* | 140.926 | 95.5899 | 66.7787 | 13.3689 | 26.5926 | 41.1912 | 1.844090462 | 0.0003 |
| *Rusc2* | 197.968 | 174.635 | 202.904 | 355.23 | 620.493 | 291.771 | -1.142866175 | 0.00032 |
| *Trim14* | 139.249 | 105.394 | 83.9014 | 36.2869 | 47.5443 | 49.4294 | 1.268332732 | 0.00032 |
| *Gm43370* | 0 | 0 | 0 | 9.54918 | 14.505 | 9.61128 | -5.997987501 | 0.00032 |
| *Gm13560* | 16.777 | 39.8291 | 49.6559 | 118.41 | 90.2536 | 113.962 | -1.559958272 | 0.00033 |
| *Adgre4* | 28.5208 | 29.4123 | 25.6841 | 97.4017 | 74.1369 | 56.2946 | -1.425955731 | 0.00036 |
| *Cgn* | 25.1654 | 10.4168 | 5.13682 | 0 | 0 | 0 | 6.15618365 | 0.00036 |
| *Tns1* | 58.7194 | 40.4419 | 38.5262 | 84.0328 | 107.176 | 186.047 | -1.486618412 | 0.00041 |
| *Gm45878* | 31.8762 | 32.4761 | 22.2596 | 5.72951 | 2.41751 | 6.8652 | 2.540450931 | 0.00042 |
| *Col11a2* | 122.472 | 112.747 | 83.0453 | 53.4754 | 47.5443 | 43.2508 | 1.149865489 | 0.00042 |
| *Mug2* | 424.457 | 278.804 | 485.43 | 57.2951 | 122.487 | 216.254 | 1.567712675 | 0.00045 |
| *Gpr182* | 35.2316 | 28.7995 | 28.2525 | 76.3935 | 70.9135 | 67.279 | -1.23046746 | 0.00047 |
| *Acot1* | 0 | 1.22551 | 3.42455 | 30.5574 | 20.9517 | 12.3574 | -3.614701492 | 0.0005 |
| *Thap12* | 147.637 | 127.453 | 59.9296 | 353.32 | 264.314 | 216.254 | -1.318980346 | 0.00053 |
| *Tdrkh* | 53.6863 | 55.7608 | 68.4909 | 145.148 | 100.729 | 127.006 | -1.050629511 | 0.00055 |
| *Gm15502* | 57.0417 | 72.9179 | 62.498 | 103.131 | 135.38 | 164.765 | -1.069707997 | 0.0006 |
| *Gm49188* | 33.5539 | 34.9271 | 50.5121 | 80.2131 | 87.0302 | 109.157 | -1.21589061 | 0.0007 |
| *Olfr1039* | 45.2978 | 57.599 | 57.3612 | 1.90984 | 15.3109 | 17.163 | 2.121027224 | 0.00071 |
| *Morn3* | 30.1985 | 37.9909 | 38.5262 | 93.582 | 81.3894 | 70.025 | -1.168229771 | 0.00073 |
| *Trappc8* | 206.357 | 117.036 | 241.431 | 64.9344 | 70.9135 | 98.8589 | 1.249837631 | 0.00074 |
| *Gm14097* | 6.71079 | 17.1572 | 34.2455 | 78.3033 | 90.2536 | 59.7272 | -1.929747179 | 0.0008 |
| *Sec23a* | 0 | 0 | 0 | 0 | 25.7867 | 35.699 | -6.874481266 | 0.00083 |
| *Olfr721-ps1* | 1.6777 | 0.61276 | 0.85614 | 3.81967 | 32.2334 | 19.9091 | -4.338000077 | 0.00084 |
| *Nans* | 144.282 | 151.963 | 109.586 | 64.9344 | 78.166 | 48.7429 | 1.084414416 | 0.00086 |
| *5830462O15Rik* | 15.0993 | 14.0934 | 11.9859 | 57.2951 | 30.6217 | 48.0564 | -1.716395939 | 0.00088 |
| *Prrc2a* | 114.083 | 140.934 | 181.501 | 47.7459 | 73.331 | 78.2633 | 1.112150221 | 0.00088 |
| *Gm11770* | 0 | 32.4761 | 31.6771 | 0 | 0 | 0 | 6.844741094 | 0.00089 |
| *Necab1* | 744.897 | 564.961 | 692.615 | 187.164 | 270.761 | 468.893 | 1.100802228 | 0.00092 |
| *Gabrb1* | 16.777 | 21.4465 | 16.2666 | 68.7541 | 45.9326 | 45.3103 | -1.515640363 | 0.00092 |
| *Dnmt3l* | 135.893 | 87.0113 | 101.024 | 21.0082 | 43.5151 | 53.5486 | 1.402774449 | 0.00097 |
| *Gm19085* | 98.9841 | 175.861 | 43.663 | 305.574 | 482.695 | 216.94 | -1.653199211 | 0.00101 |
| *Gm12954* | 10.0662 | 8.57858 | 9.4175 | 0 | 0 | 0 | 5.638310135 | 0.00108 |
| *Gm21461* | 164.414 | 173.41 | 274.82 | 95.4918 | 55.6026 | 113.276 | 1.218024782 | 0.00112 |
| *Atmin* | 25.1654 | 12.2551 | 32.5332 | 0 | 4.83501 | 1.37304 | 3.319054347 | 0.00117 |
| *4930425O10Rik* | 33.5539 | 34.9271 | 51.3682 | 13.3689 | 8.86419 | 13.7304 | 1.756910967 | 0.00116 |
| *Slmap* | 68.7855 | 56.9863 | 77.0523 | 24.8279 | 31.4276 | 28.8338 | 1.231398238 | 0.00116 |
| *Zdhhc16* | 0 | 0 | 0 | 9.54918 | 12.0875 | 6.17868 | -5.713957348 | 0.00118 |
| *Mup6* | 0 | 848.054 | 2772.17 | 0 | 0 | 0 | 12.65290209 | 0.0012 |
| *Gngt1* | 18.4547 | 17.7699 | 18.835 | 3.81967 | 0 | 2.05956 | 3.447067339 | 0.00121 |
| *Gm15848* | 0 | 0 | 0 | 13.3689 | 5.64085 | 8.92476 | -5.704508021 | 0.0013 |
| *Gm37931* | 127.505 | 66.1776 | 80.4769 | 26.7377 | 36.2626 | 41.8777 | 1.342951533 | 0.00134 |
| *Smyd1* | 36.9093 | 18.9954 | 26.5402 | 7.63935 | 5.64085 | 5.49216 | 2.155107092 | 0.00145 |
| *Gtf3c6* | 0 | 0 | 0 | 9.54918 | 8.86419 | 7.55172 | -5.612276713 | 0.00149 |
| *Gm20431* | 3.35539 | 12.2551 | 14.5543 | 0 | 0 | 0 | 5.765076337 | 0.00156 |
| *Gm10873* | 16.777 | 7.35307 | 17.9789 | 36.2869 | 41.0976 | 68.652 | -1.843764332 | 0.00172 |
| *Ifi209* | 10.0662 | 10.4168 | 5.99296 | 0 | 0 | 0 | 5.555194724 | 0.00175 |
| *Gm15638* | 0 | 3.06378 | 4.28068 | 21.0082 | 18.5342 | 18.536 | -2.816214006 | 0.00183 |
| *Gm5873* | 137.571 | 89.4623 | 127.564 | 45.8361 | 40.2918 | 70.025 | 1.160535089 | 0.00187 |
| *Disp1* | 13.4216 | 4.28929 | 9.4175 | 0 | 0 | 0 | 5.573343734 | 0.00214 |
| *Bhmt* | 31.8762 | 25.7357 | 29.9648 | 51.5656 | 67.6902 | 74.8307 | -1.179440092 | 0.0022 |
| *Gm47064* | 0 | 27.574 | 23.1157 | 0 | 0 | 0 | 6.506707779 | 0.00224 |
| *Gm5844* | 18.4547 | 14.7061 | 32.5332 | 0 | 4.02918 | 4.11912 | 2.827823841 | 0.00226 |
| *Csnk1d* | 0 | 0 | 0 | 13.3689 | 7.25252 | 4.80564 | -5.561353982 | 0.00246 |
| *Gm43413* | 36.9093 | 53.3097 | 77.9084 | 164.246 | 140.215 | 91.9937 | -1.21028296 | 0.00252 |
| *U2* | 36.9093 | 59.4373 | 32.5332 | 7.63935 | 18.5342 | 9.61128 | 1.816736663 | 0.0025 |
| *Tmed7* | 15.0993 | 37.3781 | 35.9577 | 63.0246 | 89.4477 | 78.9498 | -1.352749252 | 0.00254 |
| *Pex26* | 15.0993 | 7.35307 | 4.28068 | 0 | 0 | 0 | 5.5498786 | 0.00257 |
| *Rtl1* | 5.03309 | 6.12756 | 15.4105 | 0 | 0 | 0 | 5.572404025 | 0.00257 |
| *Gm49725* | 0 | 0 | 0 | 3.81967 | 14.505 | 7.55172 | -5.632489933 | 0.00266 |
| *8030462N17Rik* | 0 | 0 | 0 | 0 | 24.9809 | 19.9091 | -6.422531251 | 0.00281 |
| *Fam207a* | 0 | 0 | 0 | 11.459 | 3.22334 | 10.9843 | -5.589322752 | 0.00287 |
| *Srpk1* | 53.6863 | 56.3735 | 69.3471 | 120.32 | 88.6419 | 175.749 | -1.10011773 | 0.00288 |
| *Pbx2* | 77.174 | 65.5649 | 156.673 | 38.1967 | 41.9034 | 37.7586 | 1.34648512 | 0.00292 |
| *Ptges3* | 50.3309 | 30.6378 | 53.9366 | 7.63935 | 15.3109 | 17.8495 | 1.653667253 | 0.00293 |
| *Olfr1443* | 30.1985 | 41.0546 | 39.3823 | 84.0328 | 93.4769 | 62.4733 | -1.091806596 | 0.00308 |
| *Lama3* | 186.224 | 98.6537 | 158.385 | 42.0164 | 44.321 | 91.3072 | 1.291127416 | 0.00311 |
| *Gm15506* | 295.275 | 338.241 | 124.996 | 687.541 | 620.493 | 381.705 | -1.154237974 | 0.00313 |
| *Mtss1* | 0 | 0 | 0 | 0 | 16.9225 | 26.7743 | -6.384552811 | 0.00321 |
| *Hba-ps4* | 0 | 8.57858 | 11.1298 | 70.6639 | 41.9034 | 18.536 | -2.631016252 | 0.00322 |
| *Gm45877* | 124.15 | 66.1776 | 15.4105 | 13.3689 | 20.1459 | 8.23824 | 2.290346273 | 0.0034 |
| *Btnl9* | 122.472 | 81.4965 | 63.3541 | 13.3689 | 33.8451 | 43.9373 | 1.494610968 | 0.00342 |
| *Bach2* | 211.39 | 111.522 | 154.105 | 80.2131 | 79.7777 | 30.8934 | 1.331040766 | 0.00346 |
| *Gm8508* | 6.71079 | 8.57858 | 3.42455 | 13.3689 | 37.0684 | 35.0125 | -2.209162944 | 0.00354 |
| *Tex50* | 0 | 0 | 0 | 0 | 25.7867 | 16.4765 | -6.335622742 | 0.00362 |
| *Gm43305* | 8.38848 | 7.96582 | 4.28068 | 19.0984 | 46.7385 | 21.2821 | -2.11149728 | 0.00373 |
| *Gm43752* | 55.364 | 58.8246 | 34.2455 | 7.63935 | 11.2817 | 24.0282 | 1.725338626 | 0.0038 |
| *Gm18225* | 33.5539 | 17.1572 | 17.1227 | 3.81967 | 4.83501 | 5.49216 | 2.194156554 | 0.00377 |
| *F830208F22Rik* | 88.9179 | 96.8154 | 71.9155 | 32.4672 | 45.1268 | 48.0564 | 1.008017178 | 0.00384 |
| *Cstb* | 0 | 0 | 1.71227 | 13.3689 | 18.5342 | 6.17868 | -4.348716709 | 0.00387 |
| *Ndufa4l2* | 45.2978 | 46.5694 | 33.3893 | 11.459 | 0 | 8.92476 | 2.68807426 | 0.00391 |
| *Gm22540* | 67.1079 | 44.1184 | 33.3893 | 17.1885 | 8.05835 | 19.9091 | 1.675093123 | 0.00391 |
| *Synpo2* | 6.71079 | 6.74031 | 8.56137 | 0 | 0 | 0 | 5.293478777 | 0.00423 |
| *Dync2i2* | 13.4216 | 6.12756 | 4.28068 | 0 | 0 | 0 | 5.382849229 | 0.00428 |
| *Eea1* | 0 | 8.57858 | 9.4175 | 32.4672 | 28.2042 | 32.953 | -2.261908698 | 0.0043 |
| *Nlrp9c* | 3.35539 | 3.06378 | 4.28068 | 21.0082 | 12.0875 | 21.2821 | -2.336658999 | 0.00432 |
| *Camk2n1* | 0 | 21.4465 | 20.5473 | 0 | 0 | 0 | 6.236134907 | 0.00443 |
| *Hip1r* | 15.0993 | 11.6424 | 16.2666 | 38.1967 | 33.8451 | 40.5047 | -1.403205811 | 0.00447 |
| *Mup-ps1* | 25.1654 | 0 | 24.828 | 0 | 0 | 0 | 6.467992417 | 0.0046 |
| *Mir6416* | 0 | 2.45102 | 1.71227 | 5.72951 | 28.2042 | 13.7304 | -3.349792372 | 0.00481 |
| *Polr2k* | 0 | 0 | 0 | 9.54918 | 4.83501 | 6.8652 | -5.312965072 | 0.005 |
| *Gm26843* | 0 | 0 | 0 | 0 | 22.5634 | 15.1034 | -6.170497824 | 0.00542 |
| *Parp6* | 0 | 18.9954 | 20.5473 | 0 | 0 | 0 | 6.149460525 | 0.00547 |
| *Gm17833* | 5.03309 | 1.22551 | 2.56841 | 26.7377 | 23.3692 | 6.8652 | -2.804982565 | 0.00558 |
| *AA388235* | 93.951 | 80.271 | 66.7787 | 28.6475 | 41.9034 | 45.3103 | 1.015756471 | 0.0056 |
| *Dhx37* | 23.4877 | 20.8337 | 0 | 0 | 0 | 0 | 6.296703508 | 0.00568 |
| *Upf1* | 36.9093 | 24.5102 | 19.6911 | 45.8361 | 83.8069 | 60.4138 | -1.276219389 | 0.00578 |
| *Coro2b* | 3.35539 | 6.12756 | 1.71227 | 11.459 | 32.2334 | 19.2226 | -2.465575386 | 0.0058 |
| *Flad1* | 31.8762 | 22.0592 | 30.8209 | 13.3689 | 3.22334 | 6.8652 | 1.956529205 | 0.00607 |
| *Grb10* | 95.6287 | 54.5353 | 74.4839 | 19.0984 | 19.3401 | 44.6238 | 1.391249963 | 0.00632 |
| *Gpat2* | 28.5208 | 0 | 17.1227 | 0 | 0 | 0 | 6.333073713 | 0.00636 |
| *Dapk2* | 0 | 475.498 | 407.521 | 0 | 0 | 0 | 10.61751391 | 0.00658 |
| *Gm11827* | 8.38848 | 4.28929 | 4.28068 | 13.3689 | 45.1268 | 17.163 | -2.24380975 | 0.00665 |
| *Ctr9* | 0 | 0 | 0 | 0 | 21.7576 | 13.7304 | -6.084880684 | 0.00672 |
| *Ly6h* | 21.8101 | 52.697 | 36.8139 | 106.951 | 109.594 | 59.0407 | -1.26778729 | 0.00683 |
| *AI838599* | 0 | 0 | 0 | 9.54918 | 6.44668 | 4.11912 | -5.228815688 | 0.00691 |
| *Nfxl1* | 21.8101 | 12.8679 | 34.2455 | 51.5656 | 81.3894 | 50.8025 | -1.428847223 | 0.00697 |
| *Septin12* | 0 | 1.83827 | 3.42455 | 11.459 | 15.3109 | 14.4169 | -2.827278675 | 0.0071 |
| *Adgrf5* | 0 | 0 | 0 | 13.3689 | 5.64085 | 2.74608 | -5.327468384 | 0.00733 |
| *Gm11843* | 20.1324 | 7.35307 | 25.6841 | 91.6721 | 51.5735 | 33.6395 | -1.73752939 | 0.00742 |
| *Gm37086* | 8.38848 | 12.2551 | 9.4175 | 21.0082 | 36.2626 | 32.953 | -1.578970595 | 0.00742 |
| *Pi4ka* | 30.1985 | 22.0592 | 13.6982 | 0 | 5.64085 | 0 | 3.41648801 | 0.0075 |
| *Ckmt1* | 0 | 0 | 0 | 0 | 16.1167 | 17.8495 | -6.022935956 | 0.00756 |
| *B830012L14Rik* | 3.35539 | 11.0296 | 11.9859 | 0 | 0.80584 | 0 | 4.60280671 | 0.00759 |
| *4930527J03Rik* | 0 | 0 | 0 | 3.81967 | 10.4759 | 5.49216 | -5.241730861 | 0.00804 |
| *Mup2* | 20533.3 | 11152.2 | 33930.4 | 85.9426 | 4769.74 | 1202.1 | 3.436968217 | 0.00824 |
| *Cyp2d12* | 6.71079 | 17.1572 | 17.1227 | 3.81967 | 1.61167 | 1.37304 | 2.778451785 | 0.00822 |
| *Ttbk2* | 0 | 17.7699 | 17.1227 | 0 | 0 | 0 | 5.970108554 | 0.00834 |
| *Sec31a* | 0 | 15.3189 | 19.6911 | 0 | 0 | 0 | 5.974110739 | 0.00832 |
| *Gm37411* | 11.7439 | 15.3189 | 20.5473 | 49.6557 | 28.2042 | 50.8025 | -1.402152529 | 0.00836 |
| *Slc25a36* | 45.2978 | 54.5353 | 47.0875 | 15.2787 | 25.7867 | 23.3417 | 1.15618978 | 0.00849 |
| *Lime1* | 50.3309 | 28.7995 | 23.9718 | 9.54918 | 13.6992 | 2.74608 | 1.996054924 | 0.00854 |
| *Herc4* | 0 | 0.61276 | 0 | 7.63935 | 12.0875 | 4.11912 | -4.5504621 | 0.0087 |
| *Got1* | 0 | 3.67653 | 0 | 17.1885 | 28.2042 | 6.8652 | -3.59395712 | 0.009 |
| *Celf3* | 26.8431 | 22.672 | 11.9859 | 36.2869 | 95.0886 | 43.2508 | -1.53686419 | 0.00903 |
| *Fez1* | 0 | 0 | 0 | 0 | 264.314 | 350.125 | -10.18236872 | 0.00916 |
| *Trim25* | 10.0662 | 17.1572 | 8.56137 | 24.8279 | 52.3793 | 32.2664 | -1.599070227 | 0.00935 |
| *Zfp119a* | 244.944 | 250.004 | 231.157 | 103.131 | 187.76 | 65.2194 | 1.024891689 | 0.00932 |
| *Zfp692* | 30.1985 | 0 | 11.1298 | 0 | 0 | 0 | 6.186489912 | 0.00935 |
| *Arfrp1* | 0 | 0 | 0 | 9.54918 | 7.25252 | 2.74608 | -5.186167995 | 0.00942 |
| *Gm45234* | 57.0417 | 0.61276 | 0 | 0 | 0 | 0 | 6.668230432 | 0.00947 |
| *Exo5* | 0 | 0 | 0 | 0 | 13.6992 | 17.8495 | -5.917191234 | 0.00976 |
| *Mrpl12* | 0 | 0 | 0 | 5.72951 | 4.83501 | 7.55172 | -5.101919243 | 0.00978 |
| *Sftpd* | 6.71079 | 4.90205 | 6.84909 | 0 | 0 | 0 | 5.032412138 | 0.00988 |
| *Cenps* | 0 | 0 | 0 | 13.3689 | 0 | 23.3417 | -6.117340149 | 0.00989 |
| *Vmn1r172* | 33.5539 | 39.2164 | 26.5402 | 9.54918 | 10.4759 | 15.79 | 1.438176289 | 0.01012 |
| *St3gal5* | 0 | 0.61276 | 3.42455 | 19.0984 | 6.44668 | 15.1034 | -3.206546209 | 0.01023 |
| *Crnde* | 18.4547 | 25.123 | 17.1227 | 34.3771 | 52.3793 | 52.1755 | -1.196328784 | 0.01072 |
| *Gm3776* | 13.4216 | 30.6378 | 17.1227 | 0 | 5.64085 | 0 | 3.328270428 | 0.01137 |
| *Ensa* | 15.0993 | 0 | 22.2596 | 0 | 0 | 0 | 6.049471446 | 0.01152 |
| *C5ar1* | 0 | 0 | 0 | 9.54918 | 3.22334 | 5.49216 | -5.085377958 | 0.01163 |
| *Tmem199* | 0 | 0 | 0 | 0 | 15.3109 | 14.4169 | -5.831505996 | 0.01172 |
| *Gm6630* | 5.03309 | 14.7061 | 11.1298 | 76.3935 | 19.3401 | 26.7743 | -1.908067821 | 0.01182 |
| *Homer3* | 20.1324 | 31.2505 | 28.2525 | 70.6639 | 40.2918 | 67.9655 | -1.131724646 | 0.01191 |
| *Dlgap1* | 10.0662 | 4.90205 | 6.84909 | 51.5656 | 22.5634 | 12.3574 | -1.99862056 | 0.01198 |
| *Gm25058* | 53.6863 | 21.4465 | 47.0875 | 11.459 | 7.25252 | 19.2226 | 1.654219693 | 0.01212 |
| *Taar7d* | 16.777 | 33.0888 | 29.9648 | 49.6557 | 59.6318 | 63.1598 | -1.080618629 | 0.01251 |
| *Gm38300* | 0 | 0 | 0 | 3.81967 | 7.25252 | 6.17868 | -5.042494013 | 0.01257 |
| *Ankrd13a* | 33.5539 | 23.8975 | 17.1227 | 0 | 9.67003 | 4.80564 | 2.226163654 | 0.01255 |
| *Idi2* | 10.0662 | 4.90205 | 3.42455 | 0 | 0 | 0 | 5.009895845 | 0.01277 |
| *Gm44949* | 10.0662 | 14.0934 | 17.9789 | 68.7541 | 20.9517 | 39.8182 | -1.569796005 | 0.01293 |
| *Zbtb39* | 45.2978 | 25.123 | 30.8209 | 63.0246 | 53.1851 | 98.1724 | -1.121738508 | 0.01291 |
| *Gm12405* | 11.7439 | 2.45102 | 5.13682 | 0 | 0 | 0 | 5.074269722 | 0.01299 |
| *Agap3* | 57.0417 | 46.5694 | 58.2173 | 30.5574 | 1.61167 | 4.80564 | 2.241162958 | 0.01307 |
| *Gm12406* | 13.4216 | 1.83827 | 5.13682 | 0 | 0 | 0 | 5.149303121 | 0.01311 |
| *Dync1li2* | 5.03309 | 6.12756 | 5.99296 | 0 | 0 | 0 | 4.936528817 | 0.01325 |
| *Enc1* | 0 | 6.12756 | 2.56841 | 15.2787 | 16.1167 | 21.2821 | -2.434835023 | 0.01345 |
| *Gm45253* | 0 | 4.28929 | 1.71227 | 21.0082 | 19.3401 | 6.8652 | -2.772670929 | 0.01354 |
| *Tor1aip2* | 0 | 1.83827 | 1.71227 | 5.72951 | 5.64085 | 26.7743 | -3.2791796 | 0.01368 |
| *Gm4777* | 38.587 | 33.0888 | 28.2525 | 45.8361 | 90.2536 | 65.2194 | -1.041923897 | 0.01403 |
| *Fam189b* | 174.48 | 129.291 | 166.947 | 45.8361 | 52.3793 | 122.887 | 1.065038593 | 0.01438 |
| *Phf24* | 10.0662 | 4.28929 | 5.13682 | 11.459 | 33.0393 | 23.3417 | -1.905793521 | 0.0145 |
| *Olfr720* | 0 | 2.45102 | 1.71227 | 1.90984 | 22.5634 | 18.536 | -3.230603568 | 0.01451 |
| *Col6a1* | 176.158 | 127.453 | 189.206 | 57.2951 | 56.4085 | 128.379 | 1.005119692 | 0.01502 |
| *Gm13301* | 0 | 0 | 0 | 5.72951 | 6.44668 | 4.11912 | -4.941726707 | 0.01529 |
| *Dcst1* | 40.2647 | 82.722 | 62.498 | 24.8279 | 32.2334 | 30.8934 | 1.080260257 | 0.01531 |
| *Ccdc103* | 0 | 3.67653 | 0 | 17.1885 | 15.3109 | 9.61128 | -3.266901899 | 0.01525 |
| *Gm37285* | 8.38848 | 6.74031 | 22.2596 | 40.1066 | 45.1268 | 28.8338 | -1.592554494 | 0.01537 |
| *Nptn* | 8.38848 | 11.0296 | 5.99296 | 0 | 0 | 1.37304 | 3.829206996 | 0.01553 |
| *Gm47431* | 0 | 1.22551 | 0 | 7.63935 | 10.4759 | 6.17868 | -3.887050984 | 0.0156 |
| *Gm12207* | 6.71079 | 11.6424 | 17.9789 | 49.6557 | 38.6801 | 21.2821 | -1.533691875 | 0.016 |
| *Icam2* | 6.71079 | 5.5148 | 4.28068 | 0 | 0 | 0 | 4.868733893 | 0.01603 |
| *Gm12397* | 6.71079 | 4.90205 | 9.4175 | 21.0082 | 20.1459 | 22.6552 | -1.614158527 | 0.01698 |
| *Gm45786* | 6.71079 | 6.12756 | 6.84909 | 13.3689 | 17.7284 | 34.326 | -1.767906893 | 0.01697 |
| *Cyp4b1* | 0 | 46.5694 | 42.8068 | 322.762 | 168.42 | 341.2 | -3.207575502 | 0.0171 |
| *Gm11525* | 11.7439 | 6.74031 | 15.4105 | 32.4672 | 28.2042 | 28.8338 | -1.416490235 | 0.01737 |
| *Cyp2d41-ps* | 45.2978 | 32.4761 | 45.3752 | 0 | 20.9517 | 4.80564 | 2.179345249 | 0.01815 |
| *Gm38070* | 3.35539 | 1.83827 | 3.42455 | 11.459 | 8.86419 | 19.2226 | -2.260064445 | 0.01833 |
| *Wbp1l* | 48.6532 | 31.2505 | 33.3893 | 40.1066 | 88.6419 | 117.395 | -1.159118098 | 0.01847 |
| *A530017D24Rik* | 15.0993 | 13.4806 | 11.9859 | 0 | 4.02918 | 0 | 3.18964801 | 0.01951 |
| *Emilin1* | 53.6863 | 53.9225 | 39.3823 | 17.1885 | 30.6217 | 20.5956 | 1.072419731 | 0.02003 |
| *Olfr1343-ps1* | 67.1079 | 55.7608 | 41.0946 | 9.54918 | 36.2626 | 15.1034 | 1.379838882 | 0.02008 |
| *Gm17936* | 36.9093 | 41.6674 | 43.663 | 21.0082 | 19.3401 | 20.5956 | 1.016070502 | 0.02026 |
| *H2bc4* | 0 | 0 | 0 | 15.2787 | 0 | 13.0439 | -5.733670325 | 0.02036 |
| *Oplah* | 0 | 13.4806 | 15.4105 | 45.8361 | 24.1751 | 50.8025 | -1.990677445 | 0.02054 |
| *Gm266* | 20.1324 | 20.8337 | 19.6911 | 11.459 | 2.41751 | 4.80564 | 1.838384064 | 0.02095 |
| *Gm13775* | 13.4216 | 3.67653 | 14.5543 | 0 | 2.41751 | 0.68652 | 3.153674898 | 0.02123 |
| *Chd6* | 0 | 0 | 0 | 0 | 12.0875 | 12.3574 | -5.550927639 | 0.02135 |
| *Gm17167* | 3.35539 | 3.06378 | 4.28068 | 13.3689 | 15.3109 | 12.3574 | -1.941181125 | 0.0215 |
| *Nxph4* | 52.0086 | 41.0546 | 57.3612 | 34.3771 | 16.1167 | 22.6552 | 1.084150884 | 0.02212 |
| *Akr1d1* | 167.77 | 251.23 | 219.171 | 208.172 | 753.456 | 328.843 | -1.016724979 | 0.02219 |
| *Cnot7* | 0 | 0 | 0 | 17.1885 | 0 | 10.2978 | -5.68562932 | 0.02259 |
| *Pde3a* | 0 | 0 | 0 | 0 | 15.3109 | 8.92476 | -5.537462057 | 0.02261 |
| *Muc1* | 78.8517 | 87.0113 | 65.9225 | 0 | 23.3692 | 37.7586 | 1.871608646 | 0.02282 |
| *Col15a1* | 97.3064 | 54.5353 | 77.0523 | 7.63935 | 40.2918 | 39.1316 | 1.338038619 | 0.02329 |
| *Map7d1* | 15.0993 | 12.2551 | 0 | 0 | 0 | 0 | 5.598131634 | 0.0239 |
| *Ogdhl* | 0 | 4.28929 | 0 | 19.0984 | 14.505 | 8.92476 | -3.083169895 | 0.02438 |
| *Cyb561d1* | 276.82 | 99.8792 | 111.298 | 66.8443 | 87.0302 | 83.7554 | 1.017394682 | 0.02435 |
| *Vps13a* | 0 | 13.4806 | 16.2666 | 47.7459 | 29.0101 | 39.1316 | -1.884174975 | 0.02465 |
| *Tax1bp1* | 0 | 0 | 53.9366 | 0 | 0 | 0 | 6.587257048 | 0.02593 |
| *Akap3* | 0 | 0 | 0 | 0 | 50.7676 | 0 | -6.590072959 | 0.02604 |
| *Rac1* | 0 | 0 | 0 | 13.3689 | 0 | 12.3574 | -5.595644691 | 0.02632 |
| *Gm38832* | 0 | 1.22551 | 0 | 5.72951 | 9.67003 | 5.49216 | -3.684848539 | 0.02812 |
| *Ankib1* | 8.38848 | 2.45102 | 7.70523 | 0 | 0.80584 | 0 | 4.046332232 | 0.02834 |
| *Gm6493* | 0 | 0 | 0 | 0 | 8.86419 | 13.0439 | -5.394183092 | 0.02958 |
| *A430110L20Rik* | 63.7525 | 69.8542 | 65.9225 | 9.54918 | 4.83501 | 45.9968 | 1.688968217 | 0.02999 |
| *Megf8* | 11.7439 | 7.96582 | 9.4175 | 22.918 | 39.4859 | 15.79 | -1.457792229 | 0.03059 |
| *Gm28856* | 10.0662 | 29.4123 | 18.835 | 30.5574 | 44.321 | 68.652 | -1.272912377 | 0.03094 |
| *Shank2* | 80.5294 | 34.9271 | 52.2243 | 13.3689 | 15.3109 | 37.0721 | 1.297176728 | 0.03104 |
| *Nid1* | 0 | 0 | 0 | 0 | 7.25252 | 14.4169 | -5.378631239 | 0.03143 |
| *4931429L15Rik* | 0 | 17.1572 | 11.9859 | 36.2869 | 42.7093 | 30.2069 | -1.825989303 | 0.03148 |
| *Rdh13* | 0 | 7.35307 | 8.56137 | 26.7377 | 24.9809 | 14.4169 | -1.940501468 | 0.03231 |
| *Ldb1* | 26.8431 | 26.9613 | 26.5402 | 80.2131 | 53.1851 | 33.6395 | -1.027063411 | 0.03274 |
| *Gm16731* | 21.8101 | 14.0934 | 17.9789 | 0 | 8.05835 | 4.11912 | 1.999729374 | 0.03294 |
| *Secisbp2l* | 0 | 7.96582 | 11.1298 | 21.0082 | 25.7867 | 26.0878 | -1.84625621 | 0.03371 |
| *Gm4887* | 43.6201 | 22.0592 | 44.5191 | 7.63935 | 12.8934 | 21.2821 | 1.329966584 | 0.03388 |
| *Mgam* | 0 | 0 | 0 | 0 | 0 | 43.9373 | -6.384265079 | 0.03398 |
| *Gm42729* | 0 | 1.22551 | 2.56841 | 17.1885 | 7.25252 | 4.80564 | -2.759971243 | 0.03414 |
| *Zfp366* | 0 | 0 | 0 | 7.63935 | 0 | 15.79 | -5.47166879 | 0.03479 |
| *Gm44215* | 5.03309 | 6.74031 | 8.56137 | 0 | 0 | 1.37304 | 3.52105914 | 0.03474 |
| *Oas2* | 15.0993 | 8.57858 | 0 | 0 | 0 | 0 | 5.385032828 | 0.0355 |
| *Gm20460* | 11.7439 | 11.6424 | 0 | 0 | 0 | 0 | 5.374111705 | 0.03568 |
| *Wdr37* | 62.0748 | 53.9225 | 84.7575 | 22.918 | 15.3109 | 50.116 | 1.162354861 | 0.03582 |
| *Rab33b* | 8.38848 | 5.5148 | 6.84909 | 19.0984 | 17.7284 | 17.8495 | -1.432045499 | 0.036 |
| *Uba7* | 16.777 | 17.1572 | 22.2596 | 24.8279 | 40.2918 | 52.862 | -1.09138845 | 0.03634 |
| *Gm26769* | 46.9755 | 17.7699 | 26.5402 | 47.7459 | 49.9618 | 95.4263 | -1.132556609 | 0.03696 |
| *Ict1os* | 0 | 0 | 0 | 0 | 5.64085 | 15.1034 | -5.316026884 | 0.0368 |
| *Gm4846* | 28.5208 | 37.9909 | 23.1157 | 38.1967 | 48.3501 | 110.53 | -1.147490791 | 0.03696 |
| *Gm49016* | 0 | 0 | 0 | 45.8361 | 0 | 0 | -6.424986115 | 0.03755 |
| *Mb* | 0 | 0 | 0 | 0 | 0 | 41.8777 | -6.315233081 | 0.03745 |
| *Zfp385a* | 0 | 9.80409 | 11.1298 | 0 | 0 | 0 | 5.235885805 | 0.03758 |
| *Csnk1e* | 26.8431 | 27.574 | 34.2455 | 0 | 10.4759 | 14.4169 | 1.729521034 | 0.03799 |
| *Ybx1* | 0 | 4.28929 | 0 | 9.54918 | 12.8934 | 14.4169 | -2.90461932 | 0.03815 |
| *Runx3* | 11.7439 | 13.4806 | 30.8209 | 32.4672 | 47.5443 | 47.3699 | -1.177486545 | 0.03885 |
| *Cd200r2* | 18.4547 | 18.3827 | 21.4034 | 43.9262 | 23.3692 | 61.1003 | -1.139679563 | 0.03913 |
| *Morn4* | 43.6201 | 36.7653 | 53.0805 | 13.3689 | 21.7576 | 28.8338 | 1.01166913 | 0.03989 |
| *Pum2* | 0 | 0 | 0 | 0 | 0 | 40.5047 | -6.267297946 | 0.04003 |
| *Ncstn* | 0 | 18.3827 | 9.4175 | 21.0082 | 70.9135 | 25.4012 | -2.018181901 | 0.04039 |
| *Cep41* | 30.1985 | 32.4761 | 37.67 | 11.459 | 17.7284 | 18.536 | 1.036290658 | 0.04056 |
| *Tmem41a* | 0 | 0 | 0 | 43.9262 | 0 | 0 | -6.363244139 | 0.04075 |
| *Fam117a* | 0 | 1.22551 | 0 | 7.63935 | 8.05835 | 3.4326 | -3.542517603 | 0.0411 |
| *4930590J08Rik* | 11.7439 | 0 | 11.1298 | 0 | 0 | 0 | 5.33592781 | 0.04153 |
| *Vps52* | 36.9093 | 28.7995 | 34.2455 | 11.459 | 21.7576 | 11.6708 | 1.120694736 | 0.04143 |
| *Mrc1* | 0 | 0 | 2.56841 | 11.459 | 4.02918 | 10.9843 | -3.278988523 | 0.04182 |
| *Slc2a4rg-ps* | 25.1654 | 15.3189 | 11.9859 | 9.54918 | 4.83501 | 2.05956 | 1.778102172 | 0.04179 |
| *Pvt1* | 20.1324 | 13.4806 | 14.5543 | 3.81967 | 4.83501 | 6.8652 | 1.562977673 | 0.04293 |
| *Gm48627* | 33.5539 | 16.5444 | 12.8421 | 5.72951 | 7.25252 | 8.23824 | 1.50376286 | 0.04371 |
| *Slc2a9* | 0 | 0 | 0 | 0 | 0 | 38.4451 | -6.192261945 | 0.04439 |
| *Usf1* | 0 | 0 | 0 | 0 | 0 | 38.4451 | -6.192261945 | 0.04439 |
| *Prss36* | 0 | 1.22551 | 4.28068 | 7.63935 | 8.86419 | 15.1034 | -2.431715425 | 0.0443 |
| *Ccdc24* | 0 | 39.8291 | 0 | 0 | 0 | 0 | 6.155022565 | 0.04477 |
| *Gm3287* | 0 | 4.28929 | 2.56841 | 11.459 | 10.4759 | 12.3574 | -2.152981278 | 0.04491 |
| *Ythdf2* | 8.38848 | 11.0296 | 10.2736 | 32.4672 | 25.7867 | 15.79 | -1.268826159 | 0.04514 |
| *Fam83h* | 0 | 0 | 0 | 11.459 | 9.67003 | 0 | -5.30679885 | 0.04516 |
| *Gm9736* | 0 | 1.22551 | 0 | 7.63935 | 6.44668 | 4.11912 | -3.467085352 | 0.04513 |
| *Gm21982* | 20.1324 | 9.80409 | 9.4175 | 5.72951 | 0 | 0 | 3.122979581 | 0.04528 |
| *4930456L15Rik* | 0 | 6.12756 | 13.6982 | 0 | 0 | 0 | 5.155190879 | 0.04536 |
| *Gm15935* | 0 | 6.74031 | 12.8421 | 0 | 0 | 0 | 5.138053484 | 0.04594 |
| *Ubash3a* | 46.9755 | 30.025 | 29.9648 | 11.459 | 22.5634 | 14.4169 | 1.090220761 | 0.04645 |
| *Pam* | 0 | 0 | 0 | 9.54918 | 11.2817 | 0 | -5.290872012 | 0.04664 |
| *N4bp2l2* | 11.7439 | 15.9316 | 20.5473 | 26.7377 | 29.8159 | 45.9968 | -1.082749091 | 0.04712 |
| *Gm9752* | 18.4547 | 10.4168 | 7.70523 | 55.3853 | 29.0101 | 14.4169 | -1.442881 | 0.04707 |
| *Sox13* | 0 | 3.06378 | 4.28068 | 11.459 | 10.4759 | 12.3574 | -2.086640635 | 0.04729 |
| *Gm13141* | 5.03309 | 12.8679 | 6.84909 | 0 | 1.61167 | 2.05956 | 2.577470899 | 0.04764 |
| *Gm12525* | 0 | 0 | 0 | 0 | 0 | 37.0721 | -6.139975973 | 0.04759 |
| *Adam15* | 0 | 0 | 0 | 0 | 0 | 37.0721 | -6.139975973 | 0.04759 |
| *Rabl6* | 8.38848 | 12.2551 | 0 | 0 | 0 | 0 | 5.199144259 | 0.04861 |
| *Gm2080* | 3.35539 | 1.22551 | 3.42455 | 9.54918 | 18.5342 | 5.49216 | -2.138768237 | 0.04871 |
| *Gm10599* | 1.6777 | 1.83827 | 2.56841 | 9.54918 | 7.25252 | 9.61128 | -2.092603623 | 0.04922 |
| *Tcf3* | 8.38848 | 14.7061 | 12.8421 | 42.0164 | 29.8159 | 17.163 | -1.242322935 | 0.04903 |
| *Jmjd1c* | 0 | 0 | 0 | 0 | 0 | 36.3856 | -6.113104025 | 0.04931 |
| *Ucma* | 18.4547 | 18.3827 | 27.3964 | 11.459 | 9.67003 | 5.49216 | 1.327298556 | 0.04949 |
| *Gm46996* | 5.03309 | 5.5148 | 8.56137 | 0 | 0.80584 | 1.37304 | 2.891651247 | 0.04961 |
| *Ndufv1* | 152.67 | 162.38 | 148.968 | 613.057 | 175.672 | 148.288 | -1.00424633 | 0.04985 |
| *Ap1m2* | 0 | 0 | 0 | 5.72951 | 4.02918 | 3.4326 | -4.625412827 | 0.03371 |
| *Gm11755* | 8.38848 | 3.06378 | 2.56841 | 0 | 0 | 0 | 4.611604684 | 0.03633 |
| *Drg1* | 0 | 0 | 0 | 7.63935 | 4.02918 | 2.74608 | -4.740200521 | 0.02798 |
| *Slc7a12* | 6.71079 | 3.06378 | 2.56841 | 0 | 0 | 0 | 4.432793593 | 0.04975 |
| *B530045E10Rik* | 0 | 0 | 0 | 7.63935 | 6.44668 | 1.37304 | -4.847256889 | 0.02776 |
| *Gm49874* | 0 | 0 | 0 | 5.72951 | 1.61167 | 5.49216 | -4.58801342 | 0.04375 |
| *6720468P15Rik* | 8.38848 | 2.45102 | 4.28068 | 0 | 0 | 0 | 4.722937091 | 0.0275 |
| *Gm46447* | 0 | 0 | 0 | 5.72951 | 3.22334 | 5.49216 | -4.763818728 | 0.02494 |
| *Dguok* | 0 | 0 | 0 | 5.72951 | 4.83501 | 2.74608 | -4.638326607 | 0.03392 |
| *Hoxd9* | 0 | 0 | 0 | 5.72951 | 5.64085 | 4.80564 | -4.931445474 | 0.01523 |
| *Ceacam19* | 6.71079 | 3.67653 | 3.42455 | 0 | 0 | 0 | 4.601235776 | 0.03277 |
| *Gm8878* | 0 | 0 | 0 | 5.72951 | 4.83501 | 4.80564 | -4.85540664 | 0.01867 |
| *Nkrf* | 8.38848 | 4.28929 | 1.71227 | 0 | 0 | 0 | 4.65422802 | 0.03588 |
| *4930566F21Rik* | 8.38848 | 3.67653 | 2.56841 | 0 | 0 | 0 | 4.676696632 | 0.0308 |
