# Supplementary material for: Impacts of Circadian Gene Period2 Knockout on Intestinal Metabolism and Hepatic Antioxidant and Inflammation State in Mice
Source: Oxid Med Cell Longev. 2022 Jul 19;2022:7896371. doi: 10.1155/2022/7896371 (PMC9325607; doi:10.1155/2022/7896371)
Supplement: Supplementary Materials — Table S1: specific primers used for RT-PCR. Table S2: the significantly differential metabolites in metabolomics data in KO and WT mice. Table S3: KEGG pathways enriched with significantly altered metabolites in KO and WT mice. Table S4: identification of DEGs in WT and KO mice in Exp1. Table S5: identification of DEGs in WT and KO mice in Exp2. [file 7896371.f1.zip › Table S5 Identification of DEGs in WT and KO mice in Exp2.docx]

Table S5. Identification of DEGs in WT and KO mice in Exp2

| Gene id | WT1 | WT2 | KO1 | KO2 | log2FoldChange | *P*-value |
| --- | --- | --- | --- | --- | --- | --- |
| *Kng1* | 3602.95 | 4240.45 | 16715.5 | 18405.1 | 2.16252 | 1E-33 |
| *Tdg-ps* | 11187.1 | 10472.3 | 2686.64 | 2912.33 | -1.952 | 4.2E-32 |
| *Gucy2d* | 1177.91 | 1315.61 | 259.15 | 247.522 | -2.2985 | 2.5E-23 |
| *Vmn1r185* | 2383.01 | 1749.34 | 550.249 | 444.486 | -2.0526 | 9.6E-16 |
| *Mdk-ps1* | 1924.46 | 1887.39 | 653.199 | 648.823 | -1.5496 | 1.2E-15 |
| *Trp53inp1* | 186.636 | 185.442 | 736.269 | 876.332 | 2.11495 | 3.7E-15 |
| *Ndufa5* | 489.457 | 423.427 | 0 | 0 | -11.486 | 1.2E-14 |
| *Dennd5a* | 353.497 | 421.366 | 0 | 0 | -11.251 | 5.2E-14 |
| *Ddb1* | 1222.41 | 1054.96 | 342.929 | 345.477 | -1.7258 | 1.2E-13 |
| *Gm15506* | 816.998 | 684.076 | 188.15 | 145.353 | -2.1654 | 1.6E-13 |
| *Hmga1b* | 452.377 | 363.673 | 44.7299 | 68.4635 | -2.8608 | 3.6E-13 |
| *Lzts2* | 1109.93 | 752.072 | 164.01 | 220.136 | -2.2802 | 1.1E-12 |
| *Igsf23* | 5585.5 | 5121.3 | 1892.15 | 2291.95 | -1.3561 | 1.7E-12 |
| *Ankrd36* | 1201.4 | 1203.31 | 383.399 | 429.74 | -1.5657 | 2E-12 |
| *Naca* | 6053.94 | 5960.94 | 2477.9 | 2815.43 | -1.183 | 1E-11 |
| *Serpinb6c* | 244.729 | 164.838 | 0 | 0 | -10.329 | 2E-11 |
| *Slc44a4* | 710.702 | 623.292 | 164.72 | 198.017 | -1.8815 | 3.2E-11 |
| *Vkorc1l1* | 6260.36 | 5474.67 | 2472.22 | 2692.19 | -1.1843 | 4.6E-11 |
| *Wnk2* | 177.984 | 154.535 | 0 | 0 | -10.029 | 8.8E-11 |
| *E130317F20Rik* | 621.71 | 728.376 | 200.93 | 207.497 | -1.7259 | 2.1E-10 |
| *Blvrb* | 401.701 | 438.88 | 87.3299 | 114.808 | -2.062 | 1.5E-09 |
| *Topbp1* | 2745.16 | 4133.3 | 8784.82 | 10084.1 | 1.45548 | 1.7E-09 |
| *Gm39329* | 27625.9 | 41097.1 | 13366.4 | 13567.4 | -1.3514 | 2.9E-09 |
| *Gm44660* | 708.23 | 615.05 | 157.62 | 219.083 | -1.8169 | 3E-09 |
| *Chchd10* | 5538.53 | 5847.61 | 2913.84 | 2483.64 | -1.0765 | 3.2E-09 |
| *Dab2* | 28.4281 | 46.3606 | 451.559 | 211.71 | 3.14459 | 3.4E-09 |
| *Hmg20b* | 4885.92 | 5015.18 | 1945.4 | 2465.74 | -1.1672 | 4.5E-09 |
| *Slamf7* | 60.5642 | 69.0257 | 307.43 | 290.706 | 2.20494 | 6.3E-09 |
| *Klf16* | 2820.56 | 2863.02 | 1264.51 | 1429.31 | -1.0778 | 7.1E-09 |
| *Gm5173* | 196.525 | 215.319 | 586.459 | 608.798 | 1.53606 | 1.9E-08 |
| *Tfpi2* | 152.028 | 104.054 | 551.669 | 445.539 | 1.96573 | 2E-08 |
| *Gm37514* | 613.058 | 478.029 | 162.59 | 173.792 | -1.6979 | 2.5E-08 |
| *Gm31036* | 70.4522 | 104.054 | 0 | 0 | -9.1005 | 2.7E-08 |
| *Dpysl5* | 866.438 | 1098.23 | 383.399 | 371.809 | -1.3796 | 4.5E-08 |
| *Cutal* | 56.8561 | 112.296 | 0 | 0 | -9.0561 | 5.3E-08 |
| *Trmt2a* | 2460.88 | 1460.87 | 519.009 | 695.168 | -1.6924 | 5.4E-08 |
| *Slc12a5* | 3546.09 | 2636.37 | 984.058 | 1360.84 | -1.3995 | 5.5E-08 |
| *Coq8b* | 98.8803 | 54.6025 | 0 | 0 | -8.9126 | 1E-07 |
| *Dlx4* | 0 | 0 | 67.4499 | 105.328 | 8.69902 | 1.2E-07 |
| *Mup-ps1* | 0 | 0 | 164.01 | 43.1847 | 8.96667 | 1.4E-07 |
| *Exoc7* | 236.077 | 329.675 | 859.809 | 757.311 | 1.51359 | 1.5E-07 |
| *Atic* | 64.2722 | 73.1467 | 0 | 0 | -8.7551 | 1.6E-07 |
| *Repin1* | 0 | 0 | 0 | 1398.76 | 25.0346 | 1.7E-07 |
| *Gm12088* | 0 | 0 | 85.9099 | 72.6766 | 8.57833 | 1.8E-07 |
| *Itch* | 3216.08 | 2446.81 | 1007.49 | 1323.98 | -1.281 | 1.8E-07 |
| *Canx* | 0 | 0 | 80.9399 | 75.8365 | 8.56125 | 1.9E-07 |
| *Tecta* | 328.777 | 347.189 | 996.838 | 766.791 | 1.3843 | 2E-07 |
| *Mrps10* | 1236 | 1207.44 | 591.429 | 519.269 | -1.1361 | 2E-07 |
| *Sbp* | 1642.65 | 1579.35 | 794.489 | 797.336 | -1.0172 | 2.1E-07 |
| *Gm15567* | 278.101 | 172.049 | 41.8899 | 38.9715 | -2.4735 | 2.6E-07 |
| *Ncoa6* | 2023.34 | 1646.32 | 837.799 | 872.119 | -1.1016 | 3.1E-07 |
| *Selenbp2* | 0 | 1131.2 | 0 | 0 | -24.466 | 3.2E-07 |
| *Vav2* | 1022.17 | 736.618 | 259.15 | 346.531 | -1.54 | 3.8E-07 |
| *Epn1* | 436.309 | 390.459 | 131.35 | 143.247 | -1.5913 | 4.2E-07 |
| *Cntnap1* | 507.997 | 350.28 | 116.44 | 130.607 | -1.7974 | 4.2E-07 |
| *Olfr767* | 636.542 | 539.843 | 198.09 | 233.829 | -1.4473 | 5E-07 |
| *Zfp872* | 594.518 | 470.817 | 163.3 | 198.017 | -1.562 | 5.1E-07 |
| *Ppif* | 961.611 | 807.704 | 361.389 | 387.609 | -1.2404 | 5.2E-07 |
| *Phrf1* | 0 | 0 | 642.549 | 0 | 23.9745 | 5.4E-07 |
| *Gm4316* | 0 | 782.979 | 0 | 0 | -23.608 | 8.1E-07 |
| *Timm29* | 412.825 | 385.308 | 144.13 | 125.341 | -1.5637 | 9.5E-07 |
| *Thpo* | 1349.72 | 1376.39 | 704.319 | 649.876 | -1.0087 | 1E-06 |
| *4932416K20Rik* | 1412.75 | 1569.05 | 766.089 | 704.647 | -1.0192 | 1E-06 |
| *Gm12996* | 0 | 0 | 0 | 372.863 | 23.2303 | 1.2E-06 |
| *Zfp933* | 561.145 | 554.266 | 234.3 | 211.71 | -1.3209 | 1.4E-06 |
| *Vpreb3* | 415.297 | 0 | 0 | 0 | -23.072 | 1.4E-06 |
| *Brms1* | 0 | 588.264 | 0 | 0 | -22.868 | 1.8E-06 |
| *Cpsf2* | 334.957 | 0 | 0 | 0 | -22.773 | 1.9E-06 |
| *Fuz* | 192.817 | 147.324 | 34.0799 | 32.6518 | -2.3474 | 2.1E-06 |
| *Scn5a* | 431.365 | 521.299 | 184.6 | 163.259 | -1.4523 | 2.2E-06 |
| *Erich6* | 0 | 0 | 0 | 411.834 | 22.5597 | 2.4E-06 |
| *Gm15153* | 1528.94 | 1085.87 | 483.509 | 596.159 | -1.2769 | 2.8E-06 |
| *Atp6v1f* | 999.927 | 1097.2 | 495.579 | 523.482 | -1.042 | 2.9E-06 |
| *Tln1* | 1013.52 | 1055.99 | 473.569 | 530.855 | -1.0442 | 3.4E-06 |
| *Fasn* | 474.625 | 370.885 | 129.22 | 157.993 | -1.56 | 3.5E-06 |
| *Zfp692* | 0 | 0 | 67.4499 | 42.1314 | 8.04646 | 3.7E-06 |
| *Olfr331* | 1290.39 | 1136.35 | 611.309 | 448.699 | -1.1925 | 3.8E-06 |
| *Rpp25l* | 446.197 | 543.964 | 198.8 | 183.271 | -1.3733 | 4.6E-06 |
| *Acbd6* | 49.4401 | 41.2094 | 0 | 0 | -8.1539 | 4.6E-06 |
| *Myo7b* | 383.161 | 372.945 | 129.93 | 148.513 | -1.4434 | 6.5E-06 |
| *Ccdc157* | 2353.35 | 1594.8 | 665.269 | 942.689 | -1.2969 | 7.2E-06 |
| *Myadml2* | 1024.65 | 1027.14 | 482.089 | 543.495 | -1.0016 | 8.2E-06 |
| *Ttpa* | 714.41 | 785.039 | 1737.37 | 1350.31 | 1.04252 | 8.4E-06 |
| *Dsg4* | 2294.02 | 1665.89 | 989.028 | 682.528 | -1.2425 | 9.1E-06 |
| *ENSMUSG00002075151* | 316.417 | 351.31 | 120.7 | 117.968 | -1.4843 | 9.5E-06 |
| *Gm12043* | 1793.44 | 1217.74 | 703.609 | 615.118 | -1.1899 | 9.6E-06 |
| *Ggt5* | 1420.17 | 1173.44 | 488.479 | 671.995 | -1.1619 | 1.1E-05 |
| *Tmem86b* | 338.665 | 264.77 | 97.9798 | 100.062 | -1.6068 | 1.2E-05 |
| *Szt2* | 0 | 0 | 43.3099 | 46.3445 | 7.75429 | 1.3E-05 |
| *Hsdl2* | 344.845 | 416.215 | 1023.82 | 748.885 | 1.2201 | 1.7E-05 |
| *Gm49288* | 266.977 | 311.131 | 88.7499 | 104.275 | -1.5862 | 1.7E-05 |
| *Nucb1* | 454.849 | 348.219 | 129.22 | 162.206 | -1.4648 | 2.2E-05 |
| *Tmc4* | 295.405 | 257.559 | 100.82 | 77.943 | -1.6238 | 2.4E-05 |
| *Sipa1* | 290.461 | 190.593 | 53.2499 | 73.7299 | -1.9263 | 2.6E-05 |
| *C030015A19Rik* | 3235.86 | 2000.72 | 1236.82 | 1130.17 | -1.1449 | 2.7E-05 |
| *Cd22* | 206.413 | 219.44 | 66.0299 | 66.3569 | -1.686 | 3.6E-05 |
| *Psenen-ps* | 530.245 | 515.117 | 249.92 | 230.669 | -1.1199 | 4.3E-05 |
| *Gm37787* | 139.668 | 147.324 | 30.53 | 37.9182 | -2.074 | 5E-05 |
| *Gm49588* | 399.229 | 396.64 | 151.23 | 180.112 | -1.2669 | 5.2E-05 |
| *Gm28382* | 490.693 | 310.101 | 124.96 | 152.726 | -1.5293 | 5.7E-05 |
| *Trim46* | 1159.37 | 800.493 | 482.089 | 358.117 | -1.2194 | 5.7E-05 |
| *Arhgef2* | 0 | 0 | 34.7899 | 35.8117 | 7.40982 | 6.7E-05 |
| *Vamp8* | 37.0801 | 25.7559 | 0 | 0 | -7.6247 | 7E-05 |
| *Fam13b* | 32.1361 | 27.8163 | 0 | 0 | -7.5576 | 9E-05 |
| *Iqcf3* | 437.545 | 427.548 | 185.31 | 204.337 | -1.1521 | 9E-05 |
| *Gm16579* | 86.5202 | 101.993 | 238.56 | 300.186 | 1.5117 | 9.7E-05 |
| *Man1a* | 0 | 0 | 39.7599 | 27.3854 | 7.33942 | 0.0001 |
| *Ifitm10* | 489.457 | 504.815 | 200.93 | 250.682 | -1.1415 | 0.0001 |
| *Gm19085* | 302.821 | 222.531 | 90.1699 | 37.9182 | -2.0241 | 0.00011 |
| *Vmac* | 579.686 | 467.727 | 232.88 | 250.682 | -1.1152 | 0.00012 |
| *Gm43123* | 217.537 | 248.287 | 78.8099 | 86.3693 | -1.4981 | 0.00013 |
| *Gm44760* | 322.597 | 332.766 | 120.7 | 147.46 | -1.2926 | 0.00014 |
| *Trim25* | 91.4642 | 109.205 | 22.01 | 17.9058 | -2.3242 | 0.00017 |
| *Gm35256* | 307.765 | 341.008 | 102.95 | 146.407 | -1.3851 | 0.00017 |
| *Akr1d1* | 257.089 | 121.568 | 48.9899 | 40.0248 | -2.0832 | 0.00018 |
| *Rnf157* | 175.512 | 161.747 | 55.3799 | 48.4511 | -1.6962 | 0.0002 |
| *Mir6418* | 70.4522 | 73.1467 | 5.67999 | 12.6394 | -3.0005 | 0.00021 |
| *Stk16* | 0 | 0 | 24.85 | 34.7584 | 7.16408 | 0.00021 |
| *Gm43485* | 86.5202 | 77.2676 | 203.06 | 264.374 | 1.51229 | 0.00023 |
| *Necab1* | 139.668 | 97.8723 | 347.189 | 282.28 | 1.41016 | 0.00023 |
| *Kifc3* | 728.006 | 305.98 | 145.55 | 180.112 | -1.6672 | 0.00024 |
| *Chmp5* | 128.544 | 188.533 | 418.899 | 362.33 | 1.29889 | 0.00025 |
| *Lmbr1l* | 0 | 0 | 26.27 | 30.5452 | 7.09576 | 0.00027 |
| *Plppr3* | 236.077 | 228.712 | 462.209 | 480.298 | 1.01991 | 0.00028 |
| *Otulin* | 390.577 | 406.943 | 198.09 | 167.472 | -1.1232 | 0.00028 |
| *Prss22* | 143.376 | 232.833 | 53.2499 | 57.9306 | -1.7619 | 0.00029 |
| *Psmd3* | 453.613 | 486.271 | 225.78 | 238.042 | -1.0199 | 0.00029 |
| *Naip3* | 0 | 0 | 24.14 | 31.5985 | 7.06756 | 0.00031 |
| *Fam3d* | 0 | 0 | 22.01 | 33.7051 | 7.0662 | 0.00033 |
| *Rhox2b* | 185.4 | 150.414 | 361.389 | 388.662 | 1.16058 | 0.00034 |
| *Thap12* | 187.872 | 169.989 | 66.0299 | 46.3445 | -1.6636 | 0.00035 |
| *Klrg1* | 103.824 | 96.8421 | 22.72 | 23.1723 | -2.1287 | 0.00036 |
| *Hmcn1* | 0 | 0 | 34.0799 | 21.0657 | 7.05587 | 0.00036 |
| *Gm20628* | 198.997 | 174.11 | 69.5799 | 53.7175 | -1.5917 | 0.00036 |
| *Spocd1* | 126.072 | 135.991 | 356.419 | 275.96 | 1.27191 | 0.00036 |
| *Rab31* | 244.729 | 225.621 | 93.0099 | 92.689 | -1.3403 | 0.00041 |
| *Ltbp4* | 255.853 | 232.833 | 528.239 | 456.072 | 1.01187 | 0.00043 |
| *Whrn* | 85.2842 | 89.6304 | 18.46 | 18.9591 | -2.2259 | 0.00051 |
| *2810029C07Rik* | 50.6761 | 49.4513 | 177.5 | 140.087 | 1.66741 | 0.00052 |
| *Slc9a1* | 182.928 | 224.591 | 72.4199 | 77.943 | -1.4407 | 0.00056 |
| *Rusc2* | 158.208 | 240.045 | 66.0299 | 69.5168 | -1.5574 | 0.00063 |
| *Serinc3* | 212.593 | 261.68 | 95.8499 | 93.7423 | -1.3234 | 0.00064 |
| *Gm43309* | 320.125 | 558.387 | 1323.44 | 787.857 | 1.26454 | 0.00066 |
| *Gm12366* | 313.945 | 327.615 | 120.7 | 162.206 | -1.1856 | 0.00078 |
| *Pvr* | 54.3841 | 85.5095 | 211.58 | 192.751 | 1.52736 | 0.0008 |
| *4930455G09Rik* | 24.7201 | 17.514 | 0 | 0 | -7.0516 | 0.00083 |
| *Hs6st1* | 344.845 | 467.727 | 180.34 | 199.071 | -1.101 | 0.00086 |
| *Ikzf3* | 197.761 | 247.256 | 78.8099 | 95.8489 | -1.3536 | 0.00094 |
| *Myl6* | 222.481 | 276.103 | 98.6898 | 109.542 | -1.2623 | 0.00094 |
| *Lnx2* | 388.105 | 361.612 | 181.76 | 191.698 | -1.0059 | 0.00098 |
| *Ppm1n* | 189.108 | 258.589 | 66.7399 | 96.9022 | -1.4589 | 0.00101 |
| *Gm49024* | 122.364 | 122.598 | 25.56 | 42.1314 | -1.8666 | 0.00101 |
| *Lime1* | 28.4281 | 7.21164 | 87.3299 | 125.341 | 2.59091 | 0.00103 |
| *Tut1* | 144.612 | 124.658 | 40.4699 | 45.2912 | -1.6525 | 0.00103 |
| *Rps7* | 207.649 | 205.017 | 480.669 | 366.543 | 1.03952 | 0.00108 |
| *Ifi47* | 161.916 | 82.4188 | 446.589 | 264.374 | 1.5462 | 0.00108 |
| *Zbtb8b* | 54.3841 | 78.2979 | 222.23 | 163.259 | 1.53711 | 0.00109 |
| *Ces2b* | 0 | 0 | 12.78 | 34.7584 | 6.83505 | 0.00109 |
| *C1ra* | 0 | 0 | 14.91 | 30.5452 | 6.77133 | 0.00119 |
| *Amh* | 378.217 | 310.101 | 160.46 | 168.525 | -1.0649 | 0.00119 |
| *Gm43413* | 189.108 | 216.349 | 44.7299 | 88.4759 | -1.6162 | 0.00129 |
| *Ceacam20* | 138.432 | 155.565 | 51.8299 | 51.6109 | -1.5075 | 0.00146 |
| *Ripor2* | 0 | 0 | 34.7899 | 10.5328 | 6.77526 | 0.00155 |
| *Cps1* | 149.556 | 188.533 | 332.989 | 367.596 | 1.04865 | 0.00163 |
| *Trappc8* | 12.36 | 18.5442 | 68.8699 | 80.0496 | 2.26078 | 0.00181 |
| *Ptbp3* | 147.084 | 176.17 | 413.929 | 291.76 | 1.12709 | 0.00184 |
| *Fam189b* | 100.116 | 90.6607 | 210.16 | 226.456 | 1.19487 | 0.00218 |
| *Rhoa* | 0 | 0 | 17.04 | 22.119 | 6.55827 | 0.00222 |
| *Gm49628* | 28.4281 | 27.8163 | 127.8 | 80.0496 | 1.88929 | 0.00226 |
| *Fermt3* | 269.449 | 252.408 | 129.22 | 115.861 | -1.0883 | 0.00229 |
| *Dixdc1* | 30.9001 | 42.2396 | 290.39 | 64.2503 | 2.27886 | 0.00233 |
| *Esp3* | 21.0121 | 23.6954 | 107.92 | 70.57 | 1.99858 | 0.00251 |
| *Gm18857* | 0 | 0 | 17.04 | 21.0657 | 6.51918 | 0.00253 |
| *Gm5893* | 0 | 0 | 19.88 | 17.9058 | 6.50864 | 0.00261 |
| *Tmem161a* | 0 | 0 | 17.75 | 20.0124 | 6.50659 | 0.00262 |
| *Lrrc45* | 364.621 | 296.708 | 148.39 | 176.952 | -1.0249 | 0.00268 |
| *Mir6415* | 102.588 | 134.961 | 37.6299 | 40.0248 | -1.6157 | 0.00275 |
| *Rab11fip3* | 102.588 | 74.1769 | 17.75 | 27.3854 | -1.9787 | 0.00283 |
| *Kif9* | 0 | 0 | 13.49 | 24.2255 | 6.50256 | 0.0029 |
| *Wdr37* | 6.18002 | 3.0907 | 41.8899 | 38.9715 | 3.14183 | 0.00304 |
| *Tesk1* | 207.649 | 143.203 | 41.1799 | 76.8897 | -1.5791 | 0.00306 |
| *Gm6852* | 194.053 | 191.624 | 92.2999 | 60.0372 | -1.3333 | 0.00307 |
| *Gbx1* | 226.189 | 236.954 | 117.15 | 89.5292 | -1.1601 | 0.00308 |
| *Atr* | 0 | 0 | 12.78 | 24.2255 | 6.47491 | 0.00323 |
| *Gm15533* | 165.624 | 177.2 | 73.8399 | 68.4635 | -1.2674 | 0.00329 |
| *Atp5a1* | 0 | 0 | 19.17 | 16.8525 | 6.43982 | 0.0033 |
| *Asap3* | 161.916 | 114.356 | 39.7599 | 54.7708 | -1.5518 | 0.00333 |
| *Olfr748* | 8.65202 | 12.3628 | 56.7999 | 55.8241 | 2.41556 | 0.00351 |
| *Gm49340* | 154.5 | 105.084 | 39.0499 | 48.4511 | -1.5714 | 0.00352 |
| *Ica1* | 18.54 | 13.3931 | 0 | 0 | -6.6483 | 0.00356 |
| *0610040B10Rik* | 92.7002 | 89.6304 | 28.4 | 26.3321 | -1.7341 | 0.00381 |
| *Plin2* | 0 | 0 | 19.17 | 15.7993 | 6.39734 | 0.00383 |
| *Celf3* | 79.1042 | 117.447 | 33.3699 | 20.0124 | -1.8712 | 0.00385 |
| *Tsc22d4* | 100.116 | 113.326 | 35.4999 | 35.8117 | -1.5825 | 0.00406 |
| *Triobp* | 168.096 | 101.993 | 38.3399 | 51.6109 | -1.5896 | 0.0042 |
| *Ppfia3* | 119.892 | 89.6304 | 27.69 | 37.9182 | -1.6807 | 0.00429 |
| *Tnxa* | 197.761 | 195.745 | 74.5499 | 97.9554 | -1.1942 | 0.00431 |
| *Ldb3* | 0 | 0 | 20.59 | 13.6927 | 6.36978 | 0.00433 |
| *Camk1d* | 37.0801 | 35.028 | 105.08 | 109.542 | 1.57395 | 0.00438 |
| *Frmd4b* | 0 | 0 | 14.91 | 18.9591 | 6.349 | 0.00447 |
| *Gm5827* | 108.768 | 81.3886 | 264.12 | 183.271 | 1.239 | 0.00447 |
| *Zfp119a* | 171.804 | 243.135 | 88.0399 | 92.689 | -1.2013 | 0.00458 |
| *Iffo2* | 128.544 | 119.507 | 51.1199 | 35.8117 | -1.5053 | 0.00461 |
| *Btnl1* | 232.369 | 162.777 | 80.2299 | 88.4759 | -1.2279 | 0.0048 |
| *Zgpat* | 40.7881 | 7.21164 | 105.79 | 128.501 | 2.29928 | 0.00507 |
| *Atg9b* | 86.5202 | 104.054 | 30.53 | 30.5452 | -1.6426 | 0.00513 |
| *Cntfr* | 60.5642 | 38.1187 | 7.09999 | 10.5328 | -2.4956 | 0.00537 |
| *Crem* | 97.6443 | 66.9653 | 222.23 | 170.632 | 1.26 | 0.00541 |
| *D830036C21Rik* | 77.8682 | 103.023 | 18.46 | 32.6518 | -1.8368 | 0.0055 |
| *Celf1* | 32.1361 | 66.9653 | 152.65 | 140.087 | 1.55659 | 0.00558 |
| *Pcdhgb4* | 22.2481 | 30.907 | 99.3998 | 80.0496 | 1.75265 | 0.00566 |
| *Septin2* | 74.1602 | 39.1489 | 149.1 | 161.152 | 1.45842 | 0.00591 |
| *Gm39326* | 175.512 | 116.417 | 59.6399 | 51.6109 | -1.3876 | 0.00614 |
| *Polr2g* | 128.544 | 117.447 | 34.7899 | 53.7175 | -1.4826 | 0.00624 |
| *Zfp526* | 234.841 | 255.498 | 95.1399 | 136.927 | -1.0846 | 0.00626 |
| *Cbarp* | 202.705 | 199.866 | 92.2999 | 100.062 | -1.0667 | 0.00664 |
| *Abhd16a* | 0 | 0 | 14.91 | 15.7993 | 6.20863 | 0.00685 |
| *Btnl9* | 143.376 | 71.0862 | 353.579 | 196.964 | 1.36585 | 0.00753 |
| *Enho* | 110.004 | 161.747 | 33.3699 | 61.0905 | -1.5353 | 0.00754 |
| *Gemin5* | 170.568 | 165.868 | 58.9299 | 85.316 | -1.2277 | 0.00755 |
| *Cep20* | 0 | 0 | 16.33 | 13.6927 | 6.17721 | 0.0076 |
| *Gm48772* | 24.7201 | 31.9373 | 104.37 | 76.8897 | 1.67684 | 0.00763 |
| *Gm11914* | 25.9561 | 27.8163 | 104.37 | 70.57 | 1.70402 | 0.00794 |
| *Itga7* | 192.817 | 250.347 | 110.05 | 104.275 | -1.0484 | 0.00794 |
| *Gm10648* | 170.568 | 145.263 | 67.4499 | 71.6233 | -1.1836 | 0.00815 |
| *Dnah2os* | 102.588 | 69.0257 | 24.85 | 28.4387 | -1.6885 | 0.00841 |
| *Smg9* | 0 | 0 | 13.49 | 15.7993 | 6.13981 | 0.00843 |
| *Ttbk2* | 0 | 0 | 10.65 | 18.9591 | 6.15346 | 0.00855 |
| *Frmd6* | 0 | 0 | 16.33 | 12.6394 | 6.12609 | 0.00889 |
| *Mamdc4* | 348.553 | 194.714 | 122.12 | 126.394 | -1.1269 | 0.0089 |
| *Mbd6* | 32.1361 | 83.449 | 186.02 | 149.566 | 1.53219 | 0.00913 |
| *Gm19174* | 92.7002 | 124.658 | 46.1499 | 24.2255 | -1.6168 | 0.00922 |
| *Micall1* | 0 | 0 | 14.91 | 13.6927 | 6.10684 | 0.00927 |
| *Gm10323* | 126.072 | 120.537 | 47.5699 | 52.6642 | -1.3006 | 0.0093 |
| *Pde4dip* | 156.972 | 147.324 | 62.4799 | 72.6766 | -1.1733 | 0.00956 |
| *Rab11b* | 152.028 | 191.624 | 77.3899 | 80.0496 | -1.1278 | 0.00962 |
| *Mmp24* | 102.588 | 81.3886 | 32.6599 | 30.5452 | -1.5388 | 0.00963 |
| *AA388235* | 216.301 | 126.719 | 65.3199 | 75.8365 | -1.2812 | 0.00964 |
| *Scp2* | 16.068 | 18.5442 | 75.2599 | 54.7708 | 1.9099 | 0.00981 |
| *Iffo1* | 161.916 | 139.082 | 65.3199 | 68.4635 | -1.1699 | 0.01014 |
| *Gm11972* | 97.6443 | 93.7514 | 37.6299 | 30.5452 | -1.4845 | 0.01016 |
| *Gm5724* | 88.9922 | 106.114 | 227.2 | 180.112 | 1.0621 | 0.01025 |
| *Wdr91* | 0 | 0 | 14.2 | 13.6927 | 6.07033 | 0.01027 |
| *Tnfrsf1b* | 16.068 | 9.27211 | 0 | 0 | -6.314 | 0.01029 |
| *Hmces* | 180.456 | 273.012 | 118.57 | 91.6357 | -1.1074 | 0.01038 |
| *Mug1* | 105.06 | 203.987 | 317.37 | 338.104 | 1.08094 | 0.01041 |
| *Zfp385c* | 35.8441 | 12.3628 | 93.7199 | 84.2627 | 1.89574 | 0.01058 |
| *Gm23472* | 42.0241 | 44.3001 | 111.47 | 109.542 | 1.35581 | 0.01073 |
| *Notch4* | 8.65202 | 16.4838 | 0 | 0 | -6.3058 | 0.0108 |
| *Col15a1* | 84.0482 | 61.8141 | 187.44 | 144.3 | 1.19017 | 0.01119 |
| *Gm43004* | 152.028 | 114.356 | 58.2199 | 52.6642 | -1.2613 | 0.01144 |
| *1700047K16Rik* | 0 | 0 | 15.62 | 11.5861 | 6.0357 | 0.01153 |
| *Gm10505* | 149.556 | 129.81 | 58.2199 | 64.2503 | -1.1908 | 0.01159 |
| *Keap1* | 25.9561 | 25.7559 | 121.41 | 55.8241 | 1.78179 | 0.01161 |
| *F830208F22Rik* | 45.7321 | 48.421 | 110.76 | 122.181 | 1.30536 | 0.01174 |
| *Baz1b* | 159.444 | 154.535 | 73.8399 | 72.6766 | -1.0992 | 0.01201 |
| *Platr29* | 0 | 0 | 12.78 | 13.6927 | 5.9944 | 0.01267 |
| *Lingo3* | 80.3402 | 58.7234 | 141.29 | 172.739 | 1.17655 | 0.01286 |
| *Gm5717* | 32.1361 | 27.8163 | 106.5 | 70.57 | 1.56731 | 0.01347 |
| *Arhgef18* | 0 | 0 | 6.38999 | 21.0657 | 6.04229 | 0.0136 |
| *Trim33* | 86.5202 | 108.175 | 41.1799 | 29.492 | -1.4565 | 0.01371 |
| *Gm37163* | 87.7562 | 128.779 | 242.11 | 198.017 | 1.02176 | 0.01536 |
| *Wdfy2* | 39.5521 | 36.0582 | 7.80999 | 7.37299 | -2.3137 | 0.01558 |
| *E330040D14Rik* | 27.1921 | 24.7256 | 75.9699 | 76.8897 | 1.55906 | 0.01562 |
| *Hcar2* | 111.24 | 106.114 | 36.9199 | 50.5576 | -1.3188 | 0.01564 |
| *Arhgap12* | 21.0121 | 31.9373 | 95.8499 | 67.4102 | 1.62216 | 0.01571 |
| *Hba-x* | 0 | 0 | 11.36 | 13.6927 | 5.91426 | 0.01573 |
| *Nup85* | 200.233 | 158.656 | 90.8799 | 85.316 | -1.0242 | 0.0158 |
| *Pcolce* | 139.668 | 127.749 | 66.0299 | 52.6642 | -1.1674 | 0.01623 |
| *Gltp* | 9.88803 | 12.3628 | 0 | 0 | -6.1287 | 0.01642 |
| *Clk2* | 113.712 | 122.598 | 51.8299 | 50.5576 | -1.2066 | 0.01658 |
| *Ndufv1* | 216.301 | 190.593 | 68.1599 | 121.128 | -1.1106 | 0.01694 |
| *Mdfic* | 45.7321 | 33.9978 | 108.63 | 94.7956 | 1.35577 | 0.01708 |
| *Hpgds* | 0 | 0 | 17.75 | 7.37299 | 5.9233 | 0.01722 |
| *Itgb5* | 0 | 0 | 12.78 | 11.5861 | 5.87563 | 0.01737 |
| *Pfdn5* | 63.0362 | 61.8141 | 19.88 | 18.9591 | -1.6833 | 0.01766 |
| *Car15* | 1.236 | 56.6629 | 0 | 2.10657 | -4.8892 | 0.01782 |
| *4932443L11Rik* | 25.9561 | 32.9675 | 2.84 | 6.31971 | -2.7157 | 0.01907 |
| *Brcc3* | 86.5202 | 80.3583 | 189.57 | 149.566 | 1.0257 | 0.0191 |
| *Gm30849* | 60.5642 | 56.6629 | 13.49 | 21.0657 | -1.7728 | 0.01956 |
| *Pcolce2* | 35.8441 | 55.6327 | 116.44 | 108.488 | 1.29408 | 0.01973 |
| *Smc2* | 4.94401 | 0 | 27.69 | 28.4387 | 3.55165 | 0.02027 |
| *Car5b* | 11.124 | 13.3931 | 48.9899 | 46.3445 | 1.95673 | 0.02031 |
| *Cops7a* | 2892.25 | 169.989 | 138.45 | 110.595 | -3.6198 | 0.02034 |
| *Pik3r3* | 107.532 | 101.993 | 42.5999 | 46.3445 | -1.2375 | 0.02044 |
| *Sorbs1* | 0 | 5.15117 | 26.98 | 30.5452 | 3.43675 | 0.02052 |
| *Arhgap29* | 0 | 0 | 10.65 | 12.6394 | 5.80904 | 0.02052 |
| *Gm28967* | 81.5762 | 18.5442 | 117.15 | 207.497 | 1.70244 | 0.02062 |
| *Slx1b* | 80.3402 | 87.57 | 29.11 | 34.7584 | -1.3987 | 0.02087 |
| *Gm49319* | 0 | 0 | 14.91 | 8.42627 | 5.81563 | 0.02114 |
| *Usp42* | 0 | 0 | 11.36 | 11.5861 | 5.7884 | 0.02154 |
| *Plk3* | 168.096 | 139.082 | 68.1599 | 81.1029 | -1.0431 | 0.02159 |
| *Ctr9* | 13.596 | 7.21164 | 0 | 0 | -6.0294 | 0.02185 |
| *Pptc7* | 76.6322 | 87.57 | 147.68 | 184.325 | 1.01266 | 0.02191 |
| *Scx* | 12.36 | 8.24188 | 0 | 0 | -6.0157 | 0.02201 |
| *Angptl2* | 6.18002 | 4.12094 | 40.4699 | 22.119 | 2.6155 | 0.02204 |
| *Slc22a26* | 84.0482 | 48.421 | 22.01 | 18.9591 | -1.6873 | 0.02205 |
| *Gm12153* | 0 | 0 | 15.62 | 7.37299 | 5.79501 | 0.02291 |
| *Mib2* | 169.332 | 127.749 | 68.8699 | 73.7299 | -1.0586 | 0.02298 |
| *3110021N24Rik* | 181.692 | 121.568 | 78.8099 | 60.0372 | -1.1214 | 0.0236 |
| *Gm11340* | 6.18002 | 15.4535 | 46.8599 | 46.3445 | 2.09444 | 0.02375 |
| *Gm45137* | 28.4281 | 22.6652 | 66.7399 | 77.943 | 1.50321 | 0.02413 |
| *Rd3* | 32.1361 | 42.2396 | 101.53 | 85.316 | 1.32736 | 0.02413 |
| *Tmem179b* | 0 | 0 | 12.78 | 9.47956 | 5.7462 | 0.02419 |
| *Cibar1* | 72.9242 | 38.1187 | 141.29 | 122.181 | 1.2529 | 0.02432 |
| *B630019A10Rik* | 95.1722 | 67.9955 | 159.75 | 169.579 | 1.01567 | 0.02441 |
| *Shank2* | 6.18002 | 7.21164 | 31.24 | 35.8117 | 2.31988 | 0.02485 |
| *Phf11d* | 21.0121 | 31.9373 | 82.3599 | 68.4635 | 1.50672 | 0.02494 |
| *Tm7sf2* | 87.7562 | 81.3886 | 29.82 | 36.8649 | -1.3468 | 0.02523 |
| *Umad1* | 32.1361 | 45.3303 | 98.6898 | 92.689 | 1.30156 | 0.02544 |
| *Dpy19l3* | 7.41602 | 12.3628 | 0 | 0 | -5.9596 | 0.02555 |
| *Zfp740* | 81.5762 | 60.7839 | 23.43 | 27.3854 | -1.4881 | 0.02641 |
| *Acp7* | 134.724 | 144.233 | 67.4499 | 70.57 | -1.0163 | 0.02644 |
| *Rpusd2* | 49.4401 | 47.3908 | 117.86 | 100.062 | 1.17219 | 0.02663 |
| *Pdcd11* | 6.18002 | 13.3931 | 0 | 0 | -5.9452 | 0.0274 |
| *Gnao1* | 9.88803 | 9.27211 | 0 | 0 | -5.9121 | 0.02766 |
| *Ajm1* | 72.9242 | 99.9328 | 28.4 | 38.9715 | -1.3669 | 0.02784 |
| *Gna11* | 97.6443 | 107.144 | 42.5999 | 48.4511 | -1.1723 | 0.0294 |
| *Rock1* | 38.3161 | 69.0257 | 129.93 | 117.968 | 1.20311 | 0.02973 |
| *Parvb* | 39.5521 | 47.3908 | 107.92 | 92.689 | 1.20563 | 0.02991 |
| *Zfp236* | 0 | 0 | 9.22999 | 11.5861 | 5.64678 | 0.03015 |
| *Dpy19l4* | 27.1921 | 35.028 | 82.3599 | 76.8897 | 1.35347 | 0.03084 |
| *Gm19265* | 90.2282 | 101.993 | 48.2799 | 30.5452 | -1.2786 | 0.03108 |
| *Agbl3* | 40.7881 | 31.9373 | 98.6898 | 78.9963 | 1.29341 | 0.03112 |
| *Taf6l* | 101.352 | 118.477 | 51.8299 | 49.5044 | -1.1173 | 0.03141 |
| *Gm6543* | 42.0241 | 52.542 | 15.62 | 11.5861 | -1.7909 | 0.03149 |
| *Nolc1* | 60.5642 | 75.2072 | 27.69 | 21.0657 | -1.4728 | 0.0315 |
| *Ubtf* | 154.5 | 122.598 | 57.5099 | 76.8897 | -1.0473 | 0.03197 |
| *Dpp8* | 43.2601 | 65.935 | 129.93 | 111.648 | 1.14285 | 0.03197 |
| *Gm9298* | 21.0121 | 24.7256 | 71.7099 | 57.9306 | 1.50285 | 0.0321 |
| *Plekhg6* | 22.2481 | 29.8768 | 75.9699 | 65.3036 | 1.43619 | 0.033 |
| *Lcn10* | 84.0482 | 77.2676 | 29.82 | 35.8117 | -1.3007 | 0.03305 |
| *Olfr747* | 13.596 | 18.5442 | 58.2199 | 46.3445 | 1.6996 | 0.0332 |
| *Crkl* | 0 | 0 | 10.65 | 9.47956 | 5.60016 | 0.03341 |
| *Fktn* | 29.6641 | 40.1792 | 102.24 | 72.6766 | 1.32397 | 0.03348 |
| *Smbd1* | 0 | 0 | 8.51999 | 11.5861 | 5.59631 | 0.03384 |
| *Abcg2* | 8.65202 | 10.3023 | 43.3099 | 32.6518 | 2.00228 | 0.03503 |
| *9530046B11Rik* | 0 | 0 | 9.22999 | 10.5328 | 5.57235 | 0.03543 |
| *Nsd1* | 101.352 | 119.507 | 46.1499 | 56.8773 | -1.1046 | 0.03552 |
| *Ercc1* | 54.3841 | 57.6932 | 15.62 | 22.119 | -1.5787 | 0.03566 |
| *Sema4a* | 46.9681 | 51.5117 | 16.33 | 14.746 | -1.662 | 0.03606 |
| *Unc13d* | 66.7442 | 95.8119 | 26.98 | 37.9182 | -1.3327 | 0.03804 |
| *Gdpd3* | 33.3721 | 20.6047 | 5.67999 | 0 | -3.1826 | 0.03839 |
| *Gm14228* | 6.18002 | 14.4233 | 49.6999 | 33.7051 | 2.00856 | 0.03845 |
| *Cenpn* | 28.4281 | 0 | 0 | 0 | -6.4787 | 0.03887 |
| *Cyp2b10* | 32.1361 | 19.5745 | 0 | 5.26642 | -3.365 | 0.0389 |
| *Gm11684* | 19.7761 | 23.6954 | 55.3799 | 66.3569 | 1.48172 | 0.03919 |
| *Mid2* | 16.068 | 12.3628 | 41.1799 | 52.6642 | 1.72441 | 0.03954 |
| *B3galt9* | 0 | 0 | 10.65 | 8.42627 | 5.52323 | 0.03962 |
| *Klhl23* | 0 | 0 | 8.51999 | 10.5328 | 5.51916 | 0.03976 |
| *Ints7* | 50.6761 | 49.4513 | 129.93 | 88.4759 | 1.12853 | 0.03994 |
| *Vegfb* | 0 | 0 | 5.67999 | 13.6927 | 5.54012 | 0.04042 |
| *Gm49763* | 4.94401 | 7.21164 | 34.7899 | 24.2255 | 2.27562 | 0.04045 |
| *Kcp* | 56.8561 | 62.8443 | 22.72 | 21.0657 | -1.4497 | 0.04049 |
| *Chchd6* | 55.6201 | 111.265 | 18.46 | 40.0248 | -1.5265 | 0.04082 |
| *Cep112* | 276.865 | 0 | 0 | 0 | -9.765 | 0.0413 |
| *Ppp6r1* | 0 | 0 | 9.22999 | 9.47956 | 5.49388 | 0.0418 |
| *Eif2b2* | 0 | 0 | 9.22999 | 9.47956 | 5.49388 | 0.0418 |
| *B230206H07Rik* | 0 | 0 | 9.22999 | 9.47956 | 5.49388 | 0.0418 |
| *Muc20* | 3.70801 | 0 | 24.85 | 15.7993 | 3.50568 | 0.04218 |
| *Gm11787* | 0 | 0 | 11.36 | 7.37299 | 5.49801 | 0.04232 |
| *Emc3* | 58.0922 | 69.0257 | 22.01 | 26.3321 | -1.3996 | 0.0427 |
| *Jpt2* | 81.5762 | 96.8421 | 37.6299 | 42.1314 | -1.1646 | 0.04299 |
| *Gm48851* | 27.1921 | 0 | 0 | 0 | -6.4145 | 0.04318 |
| *Brap* | 27.1921 | 0 | 0 | 0 | -6.4145 | 0.04318 |
| *Usp45* | 51.9121 | 36.0582 | 114.31 | 84.2627 | 1.18054 | 0.0432 |
| *Tyms* | 12.36 | 10.3023 | 39.7599 | 40.0248 | 1.81853 | 0.04344 |
| *Samd9l* | 79.1042 | 61.8141 | 165.43 | 115.861 | 1.00203 | 0.04354 |
| *Tlx3* | 95.1722 | 175.14 | 66.0299 | 61.0905 | -1.0897 | 0.04355 |
| *Xpnpep3* | 3.70801 | 0 | 17.75 | 22.119 | 3.47419 | 0.04397 |
| *Gm47818* | 6.18002 | 10.3023 | 0 | 0 | -5.6966 | 0.04515 |
| *Nup155* | 6.18002 | 10.3023 | 0 | 0 | -5.6966 | 0.04515 |
| *Gm12320* | 45.7321 | 45.3303 | 95.8499 | 97.9554 | 1.08957 | 0.04596 |
| *Snx29* | 0 | 0 | 5.67999 | 12.6394 | 5.45977 | 0.04698 |
| *Ndufa4l2* | 0 | 25.7559 | 0 | 0 | -6.342 | 0.04707 |
| *Pnn* | 21.0121 | 28.8466 | 82.3599 | 51.6109 | 1.42612 | 0.04743 |
| *Gm37484* | 23.4841 | 31.9373 | 67.4499 | 71.6233 | 1.32323 | 0.04749 |
| *Gdf11* | 70.4522 | 48.421 | 23.43 | 20.0124 | -1.447 | 0.04784 |
| *Gm35082* | 39.5521 | 41.2094 | 10.65 | 13.6927 | -1.7369 | 0.0482 |
| *Gm45694* | 97.6443 | 87.57 | 43.3099 | 43.1847 | -1.0979 | 0.04843 |
| *Pdk4* | 34.6081 | 39.1489 | 69.5799 | 101.115 | 1.20614 | 0.04963 |
